# Source dynamics of Ruapehu’s 2022 volcanic unrest: insights from drumbeat seismicity, tremor, and crater lake signals

**DOI:** 10.1007/s00445-025-01823-2

**Published:** 2025-05-19

**Authors:** Liam A. Bramwell, Finnigan Illsley-Kemp, Ery C. Hughes, Sophie Butcher, Oliver D. Lamb, Yannik Behr

**Affiliations:** 1 School of Geography, Environment and Earth Sciences, Victoria University of Wellington, Wellington, New Zealand; 2 Present Address: Department of Geosciences, Gutenberg University Mainz, Mainz, Germany; 3 Te Pū Ao | GNS Science, Lower Hutt, New Zealand; 4 British Geological Survey, Edinburgh, UK; 5 Te Pū Ao | GNS Science, Taupō, New Zealand

**Keywords:** Ruapehu, Volcano seismology, Crater lake, Volcano monitoring

## Abstract

Ruapehu, one of Aotearoa New Zealand’s most active andesitic volcanoes, experienced moderate to heightened volcanic unrest beginning March 2022. This included heightened volcanic tremor, the initiation of a new heating phase at the crater lake Te Wai ā-moe, and increases in gas emissions. The unrest featured highly periodic, low-frequency earthquakes known as ‘drumbeats’. These signals have been observed around the world to often precede and/or accompany the ascent of magma and volcanic eruptions. However, Ruapehu did not erupt in 2022. In this work, approximately 43,000 discrete drumbeat events and 89 days of continuous volcanic tremor were identified over the 121-day unrest period. These were analysed in the time, amplitude, and frequency domains. We argue that increases in volcanic tremor, lake temperatures, and gas throughput are the result of magma ascent into the shallow system immediately prior to or contemporaneous with the onset of tremor. We construct a conceptual model for the generation of drumbeat, tremor, and lake temperature signals that consists of shallow magma storage, a gas cavity, a permeable cap, and the crater lake. The presence of repetitive drumbeat earthquakes results from transient sealing and failure within the fracture pathways of the permeable cap. This is driven and regulated primarily by pressure accumulation from persistently degassing magma and the strength of the sealing mechanism.

**Supplementary Information:**

The online version contains supplementary material available at 10.1007/s00445-025-01823-2.

## Introduction

Ruapehu is one of Aotearoa New Zealand’s most active volcanoes, having experienced more than 100 eruptive events of varying types and sizes over the last 135 years (Christophersen et al. 2022; Scott 2013; Leonard et al. 2021). Seismic and surficial precursors have preceded many of these eruptions, with technological advances in the last few decades allowing for a consistent monitoring network on the volcano that detects many volcanic signals (Christophersen et al. 2022). On March 21, 2022, Ruapehu’s Volcanic Alert Level (VAL; GeoNet 2024) was raised to Level 2 (moderate to heightened volcanic unrest), its highest non-eruptive state, following strong levels of volcanic tremor, the initiation of a new heating phase at the crater lake, and increases in gas emissions (GeoNet 2022b). Strong tremor continued into early May, returning to near-background levels by mid-June along with stabilising crater lake temperatures and generally lower gas emission rates until the VAL was lowered to Level 1 (minor volcanic unrest) on July 4 (GeoNet 2022f). During this unrest, tremor signals were accompanied by a series of discrete, highly periodic low-frequency (LF) events known as ‘drumbeats’, which have been observed around the world to often precede and/or accompany volcanic eruptions (Bell et al. 2017; Buurman et al. 2013; Iverson et al. 2006; Lees et al. 2008; Neuberg et al. 2000; Solórzano et al. 2023; Zobin et al. 2010). Drumbeats have previously been observed at Ruapehu in 2016, 2017, and 2018 but only lasted for a few days (S. Sherburn, *pers. communication*). Despite repeated observations at multiple localities globally, the mechanisms behind this phenomenon remain poorly understood and are yet to be examined in the context of New Zealand’s volcanoes.

**Fig. 1:**
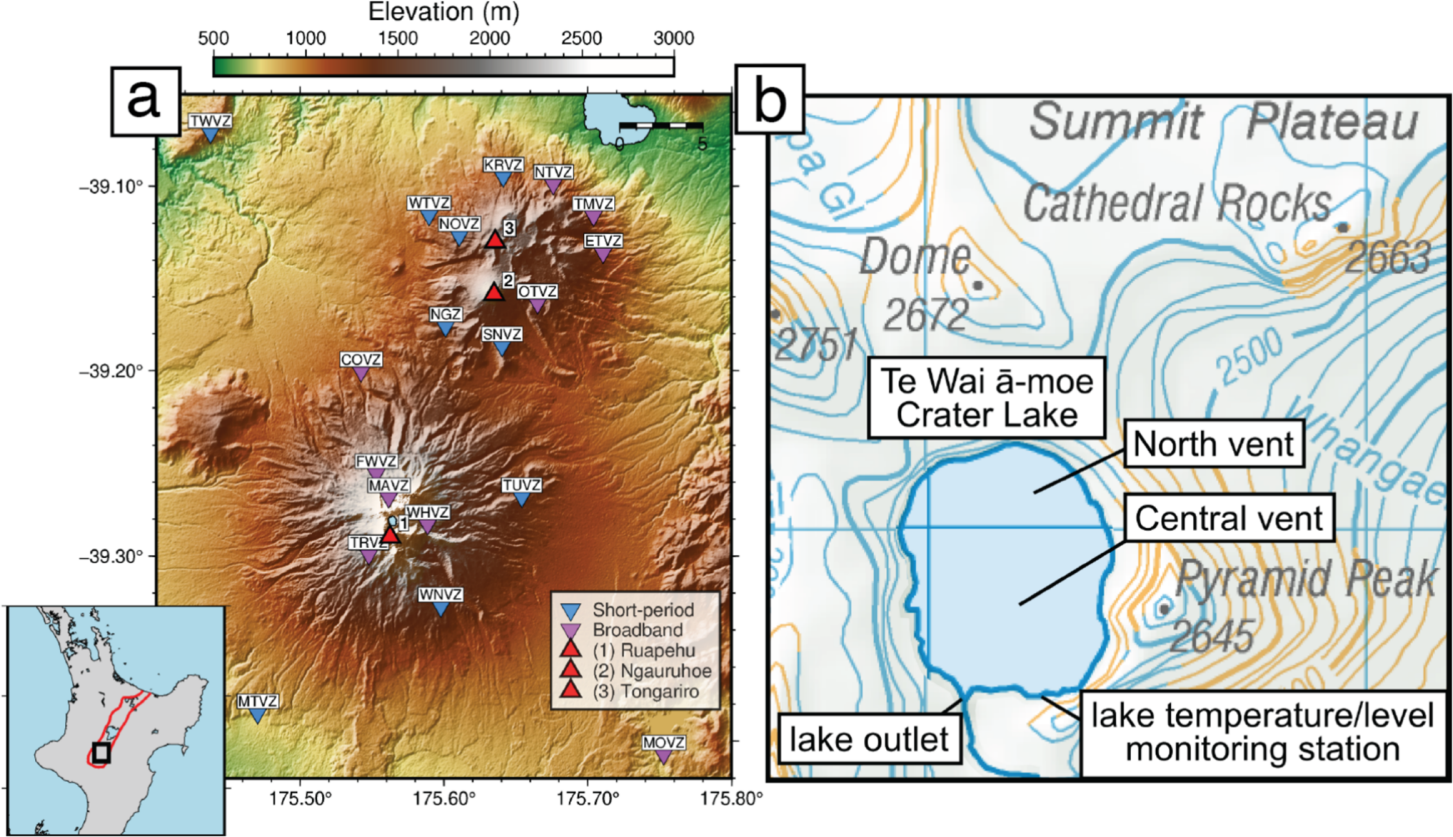
**a** Digital elevation model of Ruapehu, Ngauruhoe, and Tongariro volcanoes (red triangles), showing locations of broadband and short-period seismic stations (inverted triangles) maintained by GeoNet. The inset shows the location of Ruapehu in the North Island of New Zealand, the Taupō Volcanic Zone is outlined in red. **b** Map of the area surrounding Te Wai ā-moe Crater Lake showing the locations of the Central and North vents, GeoNet’s lake temperature and level monitoring station (GNS Science 2019), and the lake outlet. Base map (NZ Topo50 Maps, New Zealand) sourced from the LINZ Data Service licenced for reuse under CC BY 4.0

In this study, we aim to better understand the source processes behind Ruapehu’s 2022 unrest period by examining trends in seismic and lake temperature data. Specifically, we aim to integrate seismic and crater lake temperature signals to better understand the state of Ruapehu’s subsurface system at the time of this unrest, as well as determine whether these signals heralded the ascent of magma. We also aim to characterise the behaviour of discrete drumbeat earthquakes in order to determine source mechanisms, as their mere presence at Ruapehu indicates the potential for a volcanic eruption (Petersen 2007; Bell et al. 2018; Hidalgo et al. 2022). Additionally, we wish to determine the relationship between continuous tremor and drumbeat signals, as the transition between the two may indicate complex source processes.

### Geological background

Ruapehu lies at the southern end of the Taupō Volcanic Zone (TVZ; Fig. 1a), the dominant region of late-Pliocene to Quaternary volcanism in New Zealand (Wilson et al. 1995). Rhyolite is the dominant magma produced by the TVZ ($\ge$15,000 km^3^) and is largely produced by caldera-forming ignimbrite eruptions within Ahi Tupua/the central TVZ (Fig. 1a; Brown et al. 1998; Wilson et al. 1995). The central TVZ alone is the most productive and active silicic volcanic system on Earth, with a rhyolitic eruption rate of approximately 0.28 m^3^/s over the past 0.34 Ma (Wilson et al. 1995). Andesitic stratovolcanoes, like Ruapehu, are largely confined within the northern and southern TVZ. Ruapehu is a 2797 m a.s.l. (above sea level) active andesitic stratovolcano that marks the southern extent of the TVZ (Fig. 1a; Kilgour et al. 2010; Leonard et al. 2021). It is one of the largest and most active andesitic stratovolcanoes in New Zealand, being composed of several overlapping craters formed during various stages of the Holocene (Hackett and Houghton 1989). Over the last 250 ka, Ruapehu has experienced four major cone-building events from a combination of flank, summit, and satellite vents, with eruptive styles that include phreatic, phreatomagmatic, Strombolian, Vulcanian, sub-Plinian, explosive dome eruptions, and lava flow extrusions (Hackett and Houghton 1989; Kilgour et al. 2010; Doll et al. 2024). Ruapehu can be broadly divided into two components, a composite cone and the surrounding ring plain, both with volumes on the order of 150 km^3^ (Leonard et al. 2021). Ruapehu’s primary magma storage reservoir is suggested to lie $\sim$1 km north of the summit region under the volcano’s northern flank, with magnetotelluric and V_p_ anomalies and melt inclusion analyses suggesting a vertically extensive ($\sim$5 km) and $\sim$500 m wide shape extending between 2 and 7 km below the surface (Ingham et al. 2009; Kilgour et al. 2013; Leonard et al. 2021; Rowlands et al. 2005).

Over the last 5 ka, and certainly since 1830 (see Leonard et al. 2021 for summary), the entirety of Ruapehu’s volcanic activity has occurred from the summit crater, which is often topped by a crater lake (Te Wai ā-moe; Christophersen et al. 2022; Kilgour et al. 2010; Sherburn et al. 1999). Explosive eruptions occurring since the 1940s include those in 1945, 1969, 1972, 1976, 1978, 1982, 1988, 1995, 1996, and 2007, the majority of which have been phreatomagmatic or phreatic style eruptions that have ejected ash and generated lahars (Leonard et al. 2021; Sherburn et al. 1999). The exceptions to this are the 1945 major ash eruptions and lava dome emplacement that infilled the crater, and a period of initially phreatic and phreatomagmatic activity in 1995–1996 that transitioned into several distinct sub-Plinian eruptions, the extrusion of a lava spine, and intermittent ash eruptions (Christenson 2000). While little is known about the precursory signals of the 1945 eruptive sequence, the 1995–1996 sequence was well documented and several precursors were observed. In the year preceding the first explosions, Ruapehu experienced heightened seismicity in the form of shallow volcano-tectonic earthquakes and volcanic tremor with peak frequencies of 2 and 7 Hz (Bryan and Sherburn 1999). Temperatures of the crater lake fluctuated between 10 and 60 °C during this period in line with typical heating cycles, and changes in temperature did not correlate with seismicity (Bryan and Sherburn 1999). Following initial activity, changes in tremor amplitude and frequency content were often observed prior to discrete eruptions of varying types (Bryan and Sherburn 1999; Sherburn et al. 1999). Short-duration volcanic earthquakes were also recorded throughout much of the 1995–1996 unrest period. These events occurred up to 50 times per day and were characterised by low-amplitude, high-frequency onsets that were followed by higher-amplitude, low-frequency wave packets similar to the hybrid event types observed at other volcanoes (Bryan and Sherburn 1999; Lahr et al. 1994; Chouet and Matoza 2013; Moran et al. 2008). The highest density of these events occurred between November 1995 and June 1996 and were concurrent with a period of small lava extrusion and dome building (Bryan and Sherburn 1999).

Ruapehu’s most recent eruption occurred on September 25, 2007, with precursory signals which were only recognised in hindsight (Jolly et al. 2010; Kilgour et al. 2010). Ten minutes before explosive activity commenced, minor volcanic tremor, weak volcano-tectonic micro-earthquakes, and very-long period events were observed on stations near the summit (Jolly et al. 2010). As well as a steam column rising to 4500 m a.s.l, a directed Surtseyan jet produced a ballistic apron that covered an area of approximately 2.5 km^2^ in size around the summit with lapilli and ash deposits (Kilgour et al. 2010). The additional ejection of 5700 m^3^ of acidic water from the crater lake entrained roughly 60 times this volume of snow from the summit region, leading to volcanic ice-slurry flows and lahars (Lube et al. 2009). This eruption occurred during a period of seismic quiescence at the volcano, with the crater lake remaining relatively cool at $\sim$13 °C (Christenson et al. 2010; Jolly et al. 2010). In 2009, a small earthquake was followed by approximately 20 million L of extra water being added to the lake from the underlying hydrothermal system, causing a sudden 15 cm increase in crater lake level (GeoNet 2009; Ardid et al. 2023).

Ruapehu has retained a crater lake, Te Wai ā-moe, for much of recent history (Fig. 1b; Ching et al. 2024; Hurst et al. 1991). It is located in the southern region of Ruapehu’s summit area and is approximately 450 by 550 m in diameter, with a maximum depth of $\sim$130 m (Christenson 1994). The lake was first observed by western scientists in 1860 and has been present since, except during the eruptions of 1945, 1995, and 1996 when lake waters were ejected from the crater (Bryan and Sherburn 1999; Christenson 2000). Lake level is controlled primarily by a narrow outflow channel on the southern margin of the lake (Fig. 1b), the location of which has not changed since 1953 (Hurst et al. 1991). The surface often shows signs of sulphurous bubbles and slicks that float on the lake’s surface, colour variations, and upwelling sediments above subaqueous vents (Ching et al. 2024; Christenson 1994). Five active vents reside beneath the crater lake: central vent is located in the deepest and most central region of the lake; north vent in a shallower part of the lake; and three others in between (west, east, and centre-north) (Ching et al. 2024; Christenson 2000; Kilgour et al. 2010).

Ruapehu continuously degasses during times of quiescence; therefore, it is inferred that coupled convective heat and mass transfer from a shallow magmatic source to the crater lake occurs continuously (Hurst et al. 1991; Kilgour et al. 2010; Werner et al. 2006; Christenson et al. 2010). This process is not constant, however, as the lake has exhibited cyclic heating and cooling phases since at least 1986, as well as cyclic variations in gas emissions (Hurst et al. 1991; Christenson et al. 2010; Werner et al. 2006). Phases of heating generally last between 1 and 2 months while cooling phases last from 6 months to a year in length, a behaviour that Hurst et al. (1991) suggest to be the result of a liquid sulphur layer under the crater lake. It is thought that this layer acts as a natural barrier to heat transfer from depth due to liquid elemental sulphur’s rapid increase in viscosity with temperatures above 159 °C, effectively creating a cooling period by inhibiting ascending gases and heat from entering the crater lake (Hurst et al. 1991; Scolamacchia and Cronin 2016). Heating phases subsequently occur when sulphur temperatures rise above a temperature where sulphur viscosity decreases, allowing gas and heat to pass through the system more readily (Hurst et al. 1991). The observation of both cyclic heating and gas emissions may mean the sulphur seal could also be breached due to the accumulation of gas behind the seal, eventually building sufficient pressure to rupture the seal. Cyclic degassing may also be related to overpressure occurring at much greater depths (Werner et al. 2006). Ultimately, variation in gas emissions are controlled by changes in the rate of magmatic emissions (Christenson et al. 2010). It has also been suggested that heating cycles may result without the presence of a seal due to non-stationary heat flux or gas flow (Vandemeulebrouck et al. 2005). However, the relatively continuous emissions of gas from Ruapehu suggest any seal is somewhat ‘leaky’ over longer timescales. In addition to sulphur seals beneath the crater lake, the continual supply of magmatic gases and heat, alongside the presence of the crater lake and extensive hydrothermal system, can then lead to condensation of magmatic vapour and formation of other mineral seals (Christenson et al. 2010). Condensates are believed to rapidly form assemblages of sulphur, anhydrite, and natroalunite minerals that have the potential to drastically reduce permeability and effectively seal the upper portion of the vent system below the crater lake (Christenson et al. 2010). It may be that a combination of these processes is occurring at any given time.

The crater lake heating cycle before the 2022 unrest was unusually short, possibly marking the onset of a trend toward progressively shorter, more widely spaced cycles with decreasing peak heat fluxes (Behr et al. 2023). Since the unrest ended, peak lake temperatures have declined, with the last three heating cycles barely reaching 30 °C. Before 2022, many heating cycles exceeded 40 °C indicating a notable change in the processes or pathways that generate and transport heat into the lake.

### Drumbeat seismicity

Observations of drumbeat seismicity vary significantly and reports of them are relatively uncommon. Consequently, there is no consistent definition of drumbeat seismicity, nor is there a unified record of the various characteristics of drumbeats. Butcher et al. (2020) define drumbeats as swarms of periodic, highly similar repeating LFs events, with other studies adding that they are characterised by a restricted range of inter-event times (IETs) (Bell et al. 2017). However, the initial use of the term ‘drumbeats’ by Iverson et al. (2006) related to regular hybrid seismicity observed during the extrusion of a dacitic spine at Mount St. Helens volcano (USA) in 2004–2006. Observations of drumbeats at Soufrière Hills volcano (Montserrat) display similar hybrid event signals (White et al. 1998). Conversely, observations at Tungurahua volcano (Ecuador) reveal the majority of drumbeats to be LF events with dominant frequencies between 1 and 6 Hz, peaking at 3 Hz (Bell et al. 2017; Butcher et al. 2020). The variation in reported drumbeat frequency content may be due to frequency attenuation over differing source-receiver distances (Bean et al. 2014). Nevertheless, drumbeat seismicity are predominantly LF and hybrid signals within volcano-seismic observations and laboratory-controlled drumbeat generation experiments (Bell et al. 2018; Butcher et al. 2020; Kendrick et al. 2014; Moran et al. 2008). For the purposes of this study, we define drumbeats as periodic, repeating earthquakes clustered in time that display similar waveform characteristics (amplitudes, central frequencies) and regular IETs.

The variety of drumbeat observations has led to a number of source mechanisms and models being proposed. These include brittle fracturing/stick–slip shearing along conduit margins from ascending magma or during lava spine extrusion (Buurman et al. 2013; Iverson et al. 2006; Lees et al. 2008; Moran et al. 2008; Nakamichi et al. 2019; Zobin et al. 2010, 2020); gas flux and pressure variations through permeable pathways (Bell et al. 2017, 2018; Butcher et al. 2020; Garcia-Aristizabal et al. 2007; Lupi et al. 2016; Petersen 2007; Steinke et al. 2024; Voight et al. 1999); acoustic resonance of magma within the conduit (Trujillo-Castrillón et al. 2018); or rapid, repetitive venting from a shallow hydrothermal crack (Matoza et al. 2009). In this study, we will draw on different datasets as well as previous studies on the volcanic system structure to help understand the source mechanism for the drumbeats at Ruapehu in 2022.

## Data and methods

Seismic (GNS Science 2021), lake temperature/level (GNS Science 2018), and gas flux data (GNS Science 1954, 2022) for Ruapehu are recorded and maintained by GeoNet’s monitoring network. Nineteen permanent, three-component seismometers are maintained within 26 km of Ruapehu, 7 of which (5 broadbands and 2 short periods) are located $\le$9 km from Ruapehu’s crater lake (Fig. 1a). A further 4 broadband and 5 short-period stations are located on the nearby Tongariro volcano, as well as there being 3 more stations not located on any volcanoes that can still detect volcanic signals (TWVZ, MTVZ, MOVZ; Fig. 1a); see Table S2 for more details on the stations used in this study. During 2022, drumbeat signals could be observed on 15 of the 19 stations out to a distance of 21.5 km from the crater lake and volcanic conduit, with the remaining 4 stations (TMVZ, NTVZ, KRVZ, and TWVZ) either being too distal or containing a signal-to-noise ratio that was too low for events to be identified. Drumbeat seismicity was best recorded on station MAVZ located 1.45 km north of the crater lake at 2624 m elevation, and we use this station for our seismic analysis (Fig. 1a).

**Fig. 2:**
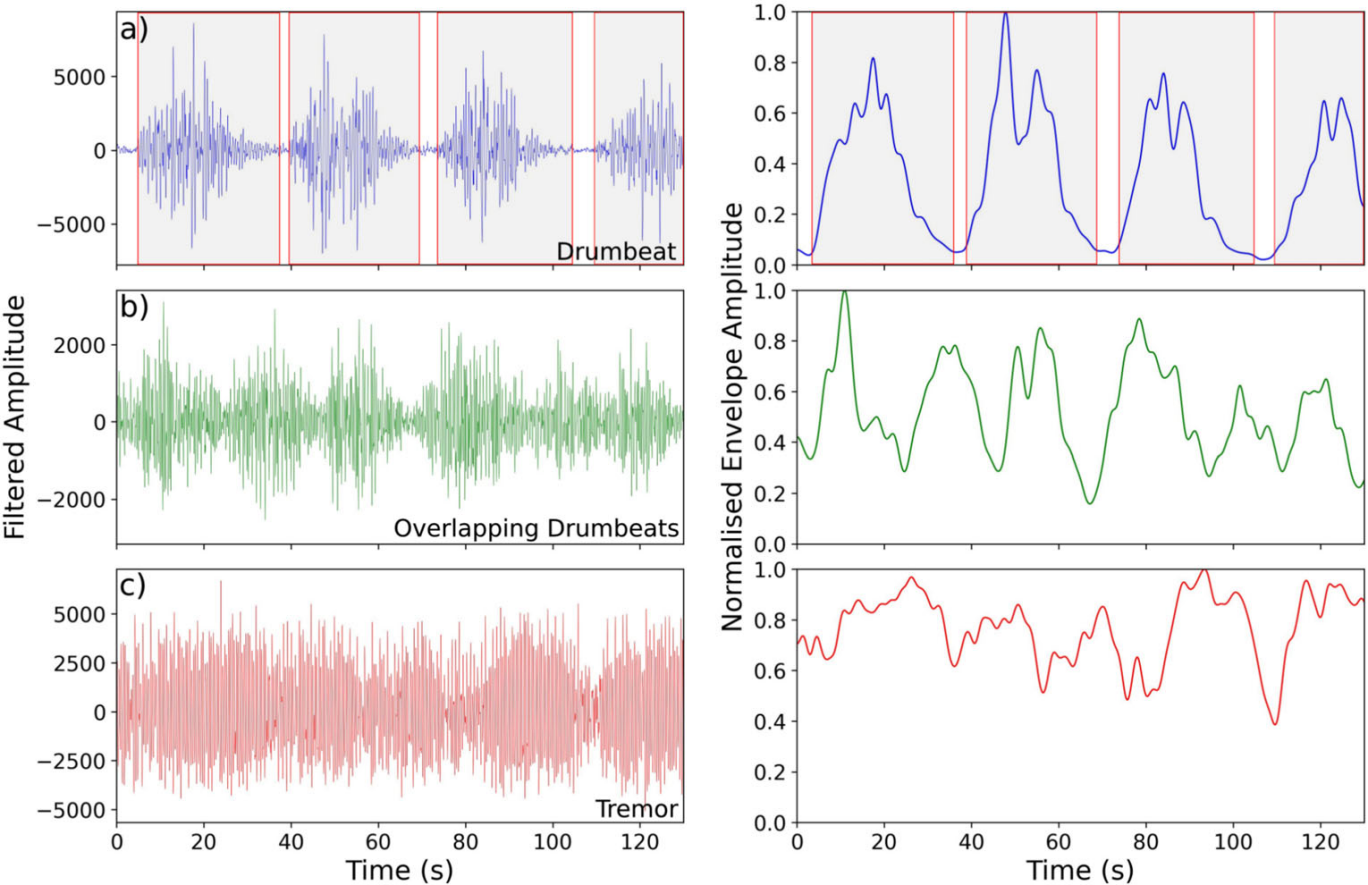
Waveform (left) and normalised envelope amplitude (right) for an example period of **a** discrete drumbeats, **b** overlapping drumbeats, and **c** tremor showing how each category was chosen. Where amplitude values approached 0 drumbeats could be distinguished and window picked (shaded boxes with red outline in **a**). However, where an adjacent event initiated during the wave packet of the previous event they could not be distinguished and form our overlapping drumbeat category (shown in **b**). Tremor was determined on the basis of sustained signal amplitudes and the lack of clear LF events (shown in **c**). All data are bandpass filtered between 0.5 and 12 Hz

Te Wai ā-moe crater lake temperatures have been measured intermittently for multiple areas of the lake since 1950 (GNS Science 1954); however, the installation of a lake temperature and level monitoring station near the lake outlet in 2008 has allowed for continuous monitoring ever since (Fig. 1b; GNS Science 2018). Temperature measurements are recorded every 15 min and transmitted several times per day via satellite to GNS Science. We also use heat emission estimates from Behr et al. (2023), who use a physics-based mass and energy balance model to estimate the heat flux into the base of Te Wai ā-moe. It assumes water is entering the lake in the form of steam from below. Inputs into the model are lake level and temperature, as well as $\text {Mg}^{2+}$ concentration of the water. This method uses a Kalman smoother and produces one estimate of heat flux per day. Ruapehu emits a gas plume through Te Wai ā-moe crater lake that is measured for SO$_{2}$, CO$_{2}$, and H$_{2}$S approximately monthly using aircraft-mounted spectrometers and electrochemical sensors (Werner et al. 2006). The monthly resolution of SO$_{2}$, CO$_{2}$, and H$_{2}$S datasets (installation around Ruapehu of the continuous ScanDOAS instruments for measuring SO$_{2}$ started in May 2022, towards the end of the 2022 unrest period) made gas flux measurements largely inapplicable to our analyses (there were only two gas flux measurements during the time periods of regular drumbeats).

All seismic events were manually picked using the Pyrocko Snuffler toolbox in the Python programming language (Heimann et al. 2017). Subsequent processing was carried out using the ObsPy Python toolkit (Krischer et al. 2015). The extent of our catalogue (March 7–July 6, 2022) covered the time when seismic signals varied from background levels. The majority of manual picking and seismic analyses used broadband station MAVZ’s vertical component (HHZ), which samples at 100 Hz. Signals were bandpass filtered between 0.5 and 12 Hz while picking to prevent obfuscation of drumbeat and tremor signals by more extreme high- and low-frequency events (e.g., volcano-tectonic events, teleseisms). Initially, we tested automatic and machine learning (ML)-based approaches to seismic event picking, including EQTransformer (Mousavi et al. 2020). From a small sample of the data, we manually inspected the quality of the automatic picks, and whilst the software detected some drumbeat signals, ultimately too many events were missed for the catalogue to be considered comprehensive. This is perhaps to be expected when using ML models pre-trained on global data, when applied in an unconventional volcano-seismic dataset (Lapins et al. 2021; Lamb 2024). Ultimately, we decided to manually pick the seismic catalogue. Three distinct types of seismicity were identified and catalogued in the catalogue: (1) discrete drumbeats, (2) overlapping drumbeats, and (3) tremor. All signals, including discrete drumbeats, were window picked over their durations to provide additional information on inter-event times and signal transitionary phases (e.g., Fig. 2a).

During picking, we encountered many instances of drumbeat signals overlapping each other and decided to pick these as periods of time. Periods of overlapping drumbeats were picked on the basis of being unable to differentiate the onset and terminus of an event from the preceding/following event due to the wave packet still entraining energy during the initiation of a new event (Fig. 2b). Tremor was defined on the basis of sustained signal amplitudes and the lack of discrete events occurring (Fig. 2c). The exact start and end of tremor windows are subject to error on the order of ±3 s due to its emergent behaviour and often transitional periods from tremor to overlapping drumbeats and vice versa.

For the drumbeat catalogue, we calculate the inter-event time (IET), measured as the time between the onsets of two consecutive drumbeat events. Rolling averages for IET measurements were calculated using a fixed 6-h window with a minimum of 50 events per window to ensure proper sampling given the length of the catalogue. Where any signal other than a drumbeat preceded a given drumbeat, that drumbeat’s IET was discounted and removed from the catalogue. We also measure each drumbeat’s central frequency, using seismic data with a high-pass filter of 0.5 Hz applied to remove the microbarom, and the same for periods of tremor. The central frequency is determined by using the method of Welch (1967) to calculate the power spectral density function of the seismic data, and using the second moment of this function to estimate where the energy is concentrated.

**Fig. 3:**
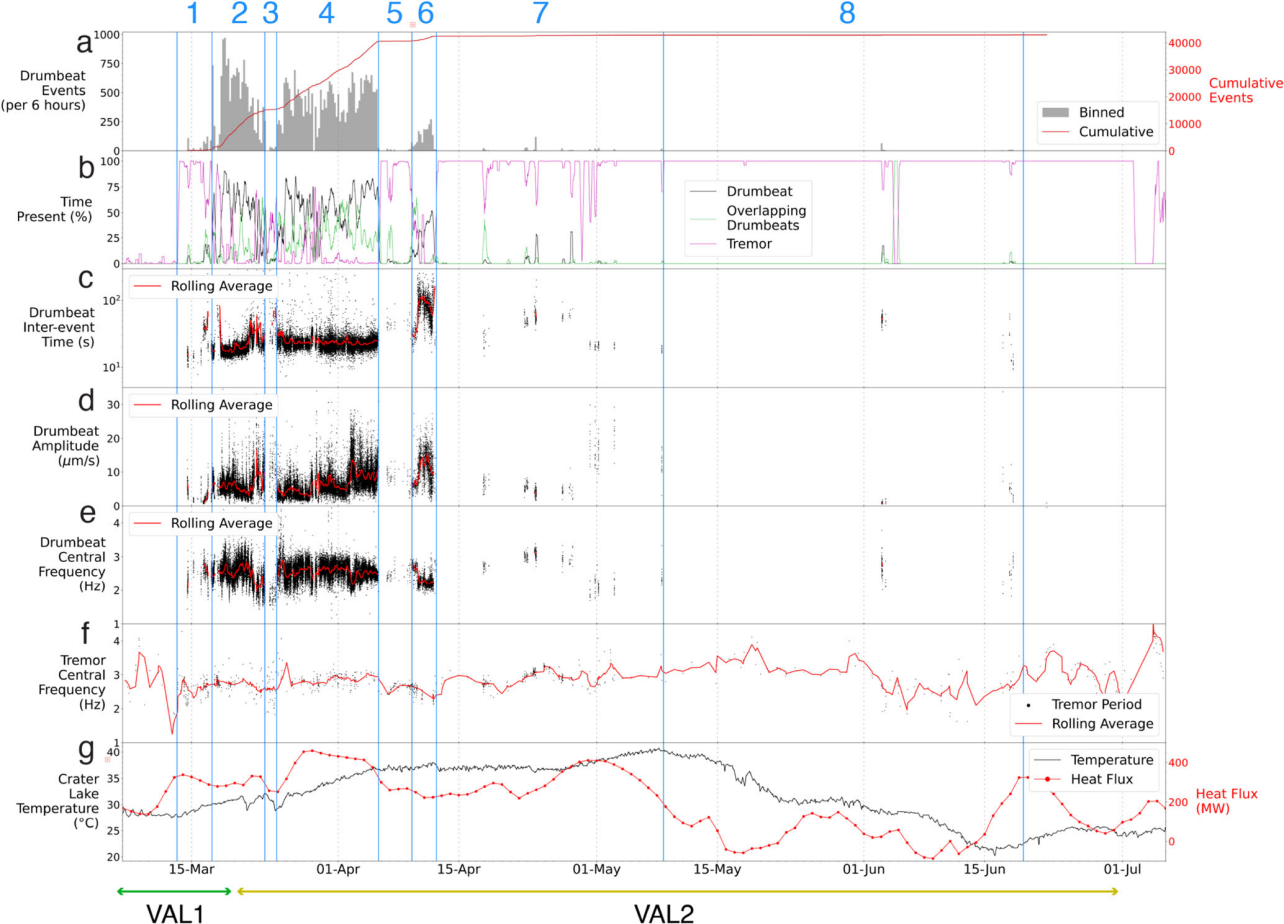
Multiple parameterisation plots of the unrest period with individual phases labelled: **a** drumbeat event rates per 6-h window with cumulative events, **b** the time each seismic signal was present for, **c** inter-event times (IETs), **d** peak to trough amplitude, **e** drumbeat central frequency, **f** tremor central frequency, and **g** crater lake temperature with heat flow model data. Rolling averages are for 6-h windows. VAL is shown across the bottom. In Fig. 4, we show a zoomed in plot of Phases 1–6

**Fig. 4:**
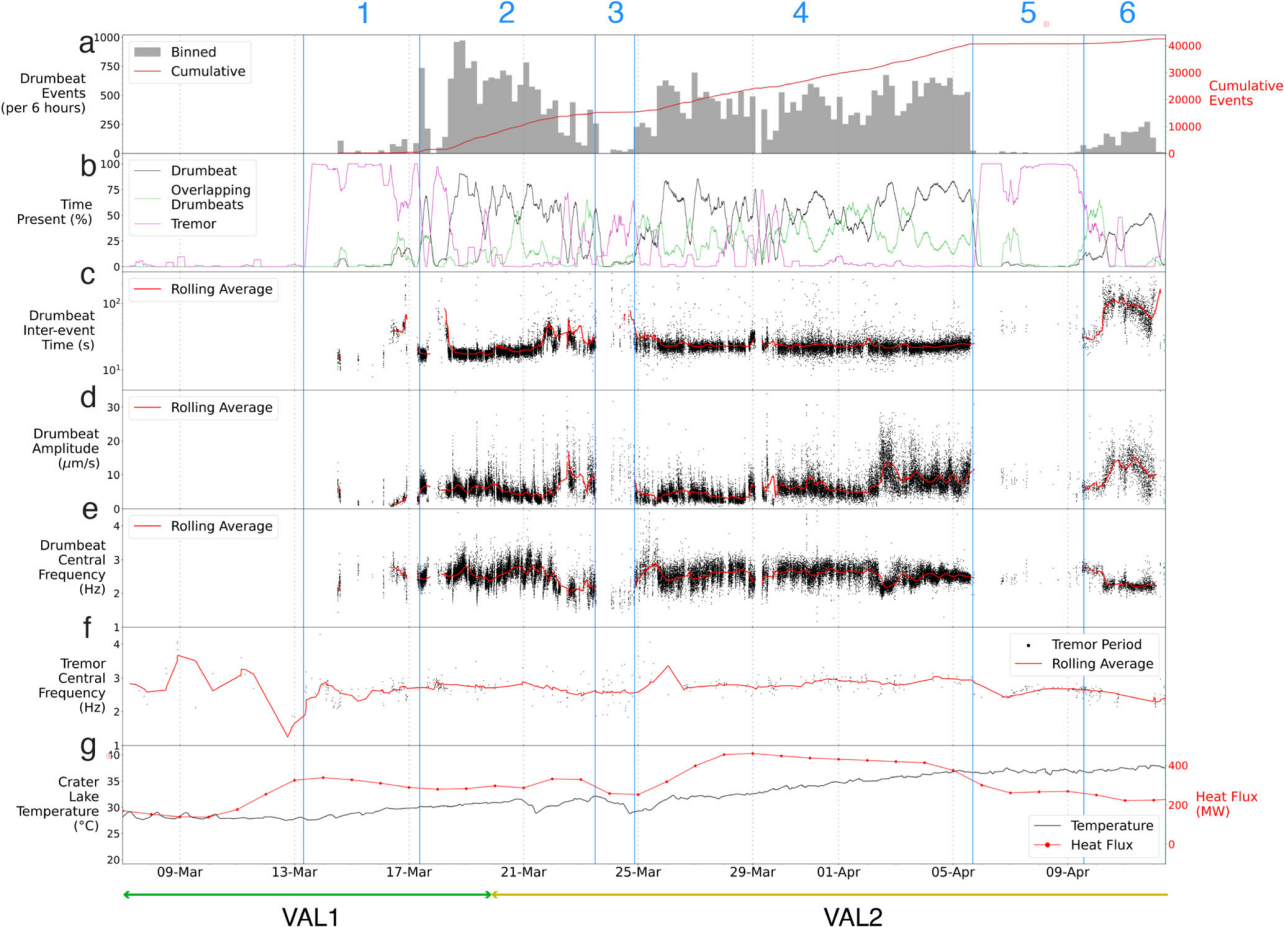
Zoomed in multiple parameterisation plots of Phases 1–6 showing, **a** drumbeat event rates per 6-h window with cumulative events, **b** the time each seismic signal was present for, **c** inter-event times (IETs), **d** peak to trough amplitude, **e** drumbeat central frequency, **f** tremor central frequency, and **g** crater lake temperature with heat flow model data. Rolling averages are for 6-h windows. VAL is shown across the bottom

**Fig. 5:**
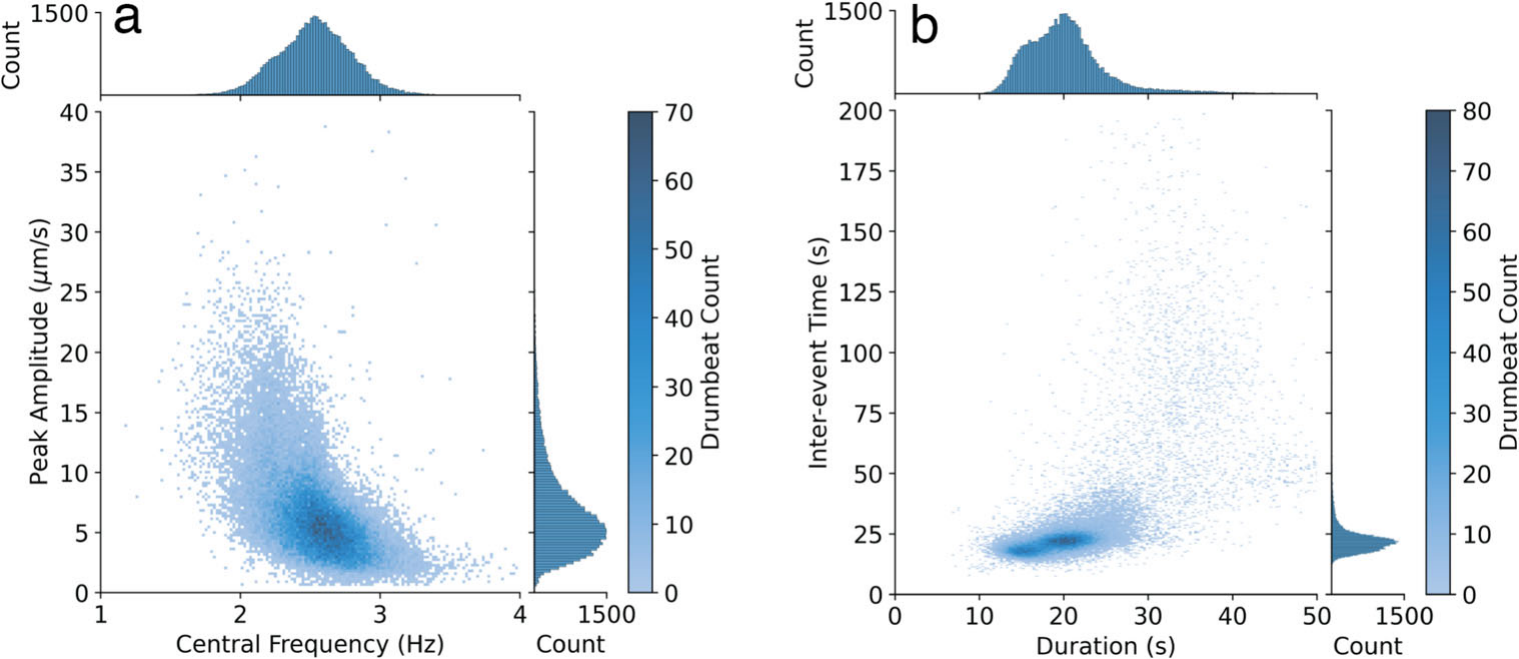
Density plot with associated histograms showing event counts for catalogue drumbeats showing the distribution of **a** peak amplitudes vs. central frequency and **b** inter-event time (IET) vs. event duration

## Results

In total, we manually picked 42,951 LF drumbeats ($\sim$10 days), $\sim$144 h of overlapping drumbeats, and >2000 h of tremor between March 7 and July 6, 2022. Almost all (98.93%) drumbeats occurred between March 14 and April 12 (Fig. 3a). Thirteen small (<100 events) drumbeat sequences were seen throughout May and June, comprising the remaining 1.07% of drumbeats observed. In total, drumbeats were extant for 8.5% of the entire 2022 unrest sequence, periods of overlapping drumbeats comprised 5.2%, and tremor 74.3% (Figs. 3b, 4b). The remaining time (12%) was exclusively periods of relative quiescence where little to no seismicity above background levels occurred. Quiescence was also present during drumbeat sequences between two events. We split the unrest into eight phases based on changes in activity, which are described in detail in the Discussion.

The majority of drumbeats displayed peak amplitudes between $0.25\times 10^{-5}$ and $2\times 10^{-5}$ m/s (Figs. 3d, 4d, 5a). In comparison, a $M_{L}$3.1 event in the Waiouru area (27 km SE of Ruapehu summit) on 4 June 2022 (GeoNet PublicID 2022p418361) had a peak amplitude of $1.8\times 10^{-5}$ m/s at MAVZ. The distribution of event amplitudes was unimodal, though with a skew towards higher amplitudes (Fig. 5a). There was a general trend of increasing drumbeat amplitude in mid-March (Phase 2) followed by a period of quiescence (Phase 3), this pattern was also seen in late March and early April (Phase 4) where drumbeat amplitudes increased in distinct steps, before another period of quiescence (Phase 5; Fig. 4d).

Broadly, drumbeat central frequencies did not span a particularly large range of frequencies; however, distinct patterns still existed (Figs. 3e, 5a). The mean peak frequency for drumbeats was 2.5 Hz, and the majority of the catalogue were between 2 and 3.2 Hz (variance of 0.08 Hz; Fig. 5a). As expected, drumbeat central frequencies were lower than 5 Hz and, as such, can be considered LF events; although drumbeat spectra can contain frequencies $\ge$5 Hz. Through time we observed a slight decrease in drumbeat frequency in March (Phase 2), whereas through late March and early April (Phase 4) drumbeat frequency is relatively constant (mean of 2.5 Hz, variance of 0.06 Hz) except for some higher frequency (>3 Hz) events in the initial stages of Phase 4 and a decrease of $\sim$0.5 Hz in April which occurred at the same time as an increase in drumbeat amplitude (Fig. 4d, e). During these same time periods, the frequency of continuous tremor was relatively constant with a mean of 2.9 Hz and variance of 0.06 Hz (Fig. 4f). During late April and May (Phase 7 and 8, Fig. 3), when tremor was the dominant signal, the tremor frequency was relatively constant at $\sim$3 Hz before gradually declining during Phase 8.

In general, drumbeat IETs had a unimodal distribution with a slight skew towards higher IETs (Fig. 5b). The majority of drumbeat IETs were centred in the 18–25 s range, with a mean of 27 s, standard deviation of 35 s, and mode of 21 s (Figs. 4c, 5a). It should be noted that there is an ‘artificial’ floor to drumbeat IETs due to how they have been calculated (i.e., time between start of two events), so IETs generally do not fall below 15 s due to signal duration length; most fall into the overlapping drumbeat category below this threshold. However, 673 events were still identified below this threshold, 85% of which display signal durations of less than 20 s. The skew towards higher IETs is caused by a smaller number of 7856 drumbeats between 25 and 200 s (Fig. 5b), and these predominantly occurred in mid-April (Phase 6; Fig. 4c).

## Discussion

In this section, we detail our interpretation of Ruapehu’s magmatic-hydrothermal system during its 2022 unrest episode. This includes a detailed description of how the observed parameters varied through time alongside our hypotheses for the processes which caused these changes. We will first outline a conceptual model for Ruapehu’s plumbing structure, supported by examples from the 2022 unrest period. We outline temporal changes in source dynamics throughout each phase of the unrest period and offer insights into signal source locations. A chronological list of observations is also detailed in Table S1.

**Fig. 6:**
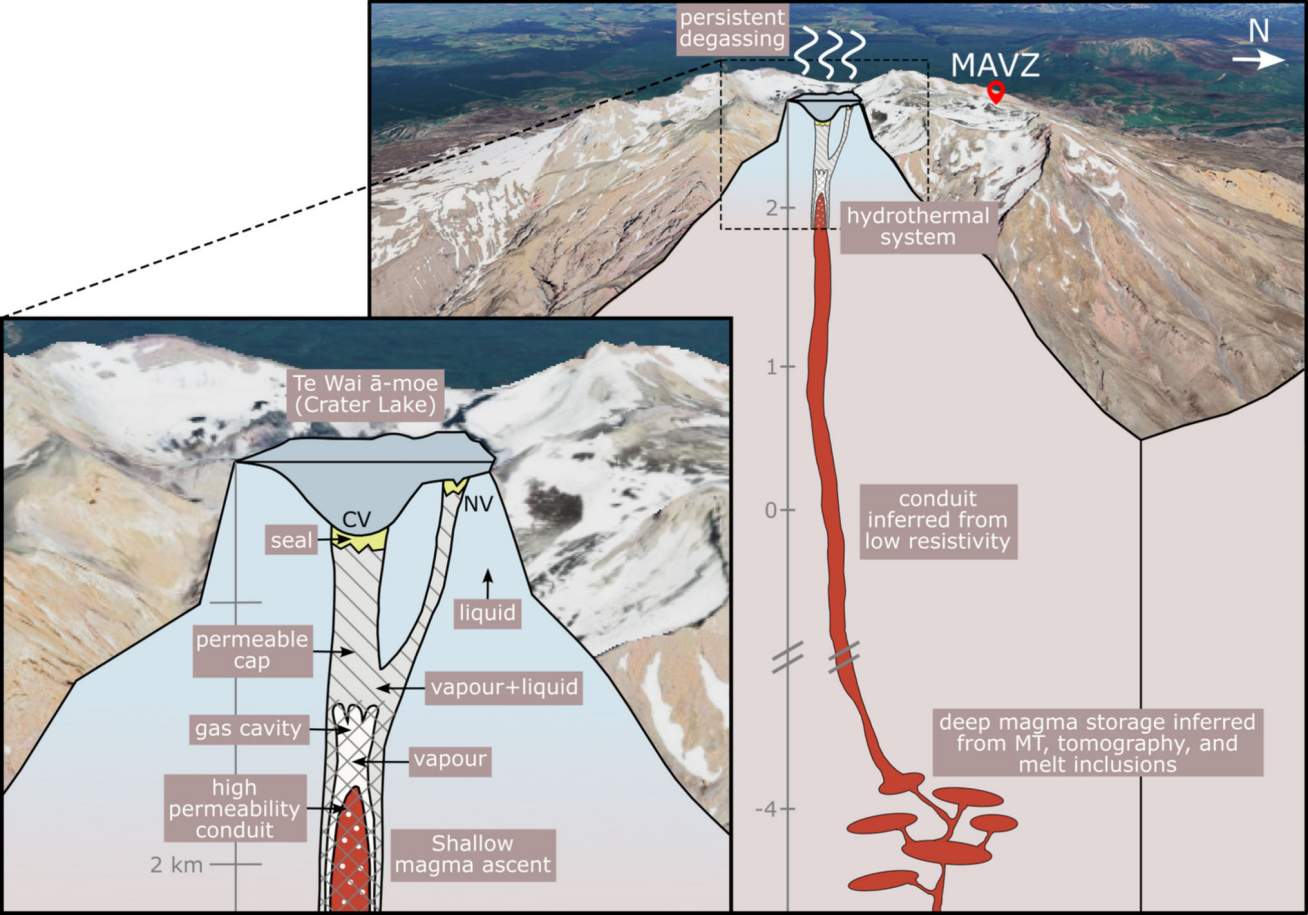
Schematic diagram of Ruapehu volcano and its subsurface magmatic-hydrothermal system. The main figure (right) illustrates the broader system, including the approximate location of deep magma storage inferred through magnetotelluric (MT) (Ingham et al. 2009), tomographic (Rowlands et al. 2005), and melt inclusion (Kilgour et al. 2013) studies. The general extent of the magmatic conduit is inferred from regions of low resistivity (Ingham et al. 2009), while the hydrothermal system is approximated using joint hydrothermal alteration and aeromagnetic data inversion analyses (Kereszturi et al. 2020). The location of seismometer station MAVZ used for seismic analysis is labelled. The inset figure (left) outlines our proposed four-component system (shallow magma ascent and storage, gas cavity, permeable cap, crater lake), alongside the central and northern vent sites (CV and NV, respectively: more vents have been observed (Ching et al. 2024) but are omitted here for simplicity) and underlying pathway geometries after Christenson et al. (2010). Background image taken from Google Earth, CNES/Airbus

### Conceptual model for Ruapehu

**Fig. 7:**
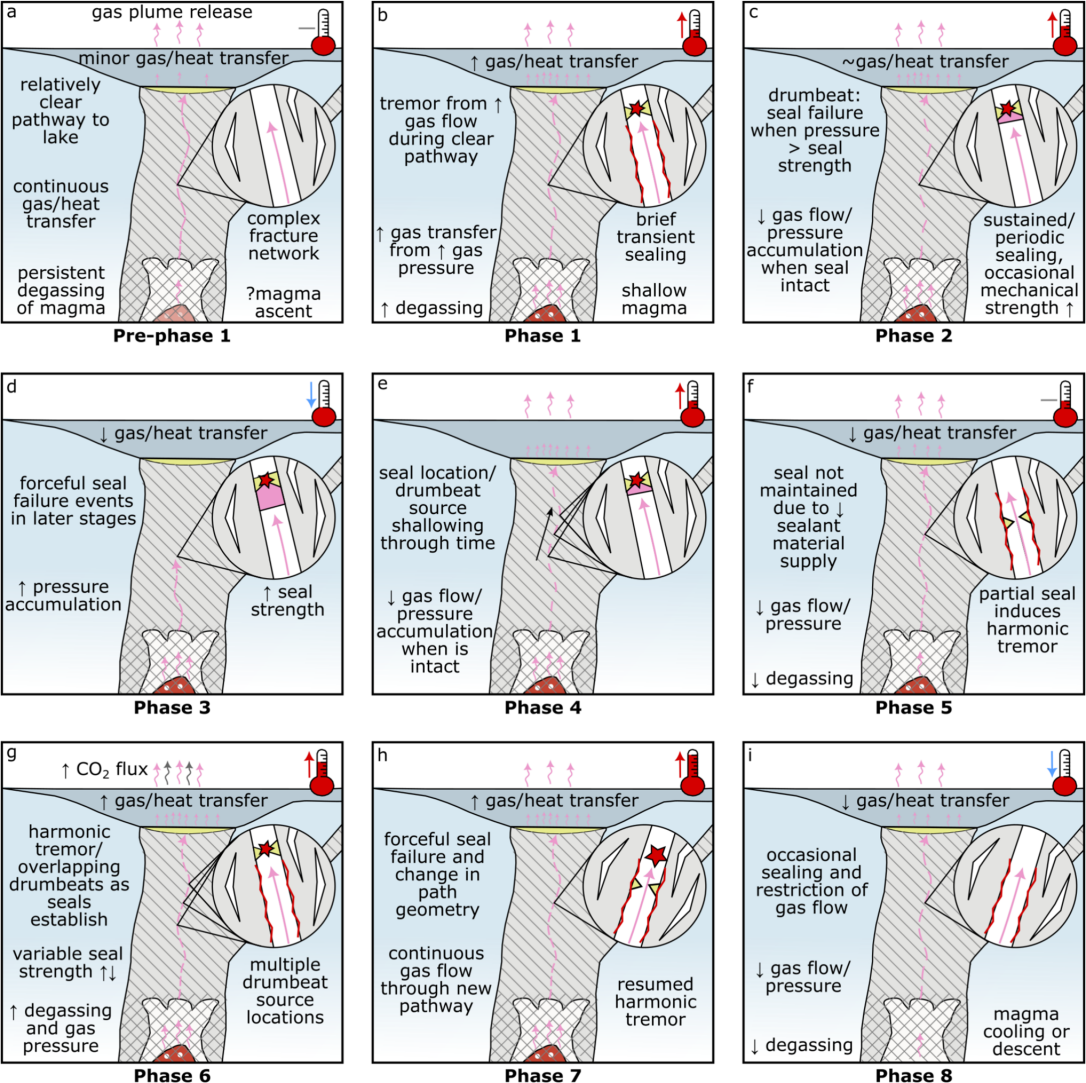
Schematic illustration showing the evolution of source processes throughout the 2022 unrest period. Vertical scale is highly compressed compared to horizontal scale, and only the central vent system is included for simplicity. The circular inset in each panel highlights the processes leading to drumbeats and tremor

By integrating previous research (Christenson et al. 2010; Werner et al. 2006; Hurst et al. 1991; Christenson and Wood 1993; Girona et al. 2018) with observations from the 2022 unrest episode, we present a conceptual model for Ruapehu’s subsurface system (Fig. 6). We will use this model to interpret changes in the observed signals through the unrest episode (Fig. 7). Based on energy mass balance observations at the crater lake during quiescence, Hurst et al. (1991) propose that all heat flux at Ruapehu is sourced from a magmatic source via a permeable pathway. This is further supported by the continuous degassing observed at Ruapehu outside of unrest episodes, with delays between degassing and lake temperature peaks suggesting non-hydrothermally sourced emissions (Christenson et al. 2010; Werner et al. 2006). In the months preceding the 2022 unrest episode, Ruapehu exhibited typical, persistent outgassing with no volcanic tremor or seismicity above expected background levels, though there was a cluster of seismicity near Ruapehu’s summit at the beginning of the year (GeoNet 2022a). This changed around 13 March, when volcanic tremor elevated above background levels and lake temperatures began increasing, subsequently followed by the onset of drumbeat seismicity (Fig. 4). The high levels of tremor, seismicity, and gas flux recorded during the Ruapehu 2022 unrest suggest magma ascended to shallow levels in the conduit to drive this energetic behaviour.

Periodic drumbeat signals require a nondestructive process with a consistent ‘loading’ rate, typically of pressure or stress accumulation; a near-constant ‘failure’ strength, which releases the pressure or stress; and a consistent slip or pressure drop (Bell et al. 2017). To explain the physical mechanism behind drumbeat seismicity during the 2022 unrest, we invoke a model which involved the loading and failure of mineral seals within a permeable fracture network in a mostly non-porous medium (permeable cap) by ascending gas (Fig. 6). We propose that, at Ruapehu, drumbeat seismicity was governed primarily by the accumulation and release of pressurised gas, as the observed emergent onsets of seismic signals precludes a shear-stress source. Hence, the ‘loading’ rate was governed by gas flow and pressurisation. This gas repeatedly broke a non-permeable seal, where the ‘failure’ strength is governed by the seal strength. The seal was consistently ‘healed’ by mineralisation (e.g., Christenson et al. 2010) allowing a nondestructive source for drumbeats. Over longer timescales than an individual drumbeat this repeated seal failure enabled high gas flux to the surface because the gas was only trapped for a short period of time. The composition of the mineral seal is difficult to constrain from our observations, but is likely sulphur-bearing such as elemental sulphur, anhydrite, or natroalunite as is commonly suggested at Ruapehu (e.g., Christenson et al. 2010).

This process was not continuous throughout the unrest sequence, as we frequently observed transitions between drumbeat seismicity and tremor and vice versa, and at no point did these two regimes occur simultaneously. This suggests that both the tremor and drumbeat seismicity shared the same changing source mechanism. We propose that tremor occurred as a result of an increased gas flux through the same permeable pathway (e.g., Iverson et al. 2006; Moran et al. 2008; Zobin et al. 2010; Yokoo et al. 2009; Butcher et al. 2020, Fig. 6).

In addition to variable gas flux through the permeable cap, we also suggest magmatic gases needed to be temporarily trapped beneath the cap before escaping through the fracture network pathway (Fig. 6). Gas cavities have previously been suggested in multiple volcanic settings, including Ruapehu (Zobin et al. 2010; Yokoo et al. 2009; Iguchi 1994; Rymer et al. 1998; Carbone et al. 2015; Tanaka et al. 2009; Vergniolle and Jaupart 1990; Girona et al. 2019) and represent where the single-phase vapour zone that occurs ahead of the magma (e.g., Christenson et al. 2010) accumulates due to a transition from a highly permeable conduit to a permeable cap (e.g., Girona et al. 2018). Under the assumption of constant seal strength mechanics, tremor signals decelerating to form overlapping drumbeats and then drumbeats are best explained by a decrease in gas flow through the pathway. Patterns such as these, as well as the reverse (drumbeats accelerating to tremor), could be explained by variable gas exsolution rates directly from shallow magma storage; a similar mechanism has been cited for low-frequency drumbeats observed at Tungurahua and Whakaari volcanoes (Butcher et al. 2020; Steinke et al. 2024). However, the timescales at which these changes occur and last for (hours to days) suggests a buffer region where exsolved gas accumulates and creates variable driving pressure conditions that control outgassing, instead of a direct route from magma to an outgassing pathway. The quantity of gas stored then dictates both the gas cavity thickness and the resulting external pressure at the exit vent, which results in changing outflow rates depending on the quantity of gas within the cavity and, in turn, the transitionary nature of 2022 unrest seismic signals.

### Evolution of source dynamics

Here, we outline changes in signal behaviour during Ruapehu’s 2022 unrest period within the context of our proposed conceptual model. Through interpretations of drumbeat event parameters (i.e., IET, amplitude, central frequency), tremor characteristics, and lake temperature/heat flux variations, we interpret distinct phases of the unrest period. We place primary focus on the key processes occurring within each phase of the unrest, including the role of ascending magma on inducing initial seismicity, variations in inferred outgassing rates (unfortunately, there were only two measurements of gas flux during Phases 1–6, Fig. S1) and seal strength, and the effect pressure accumulation at depth has on gas flux.

#### Pre-Phase 1: prior to 13 March

The period prior to Phase 1 can be described as experiencing regular ‘background’ levels of activity that are typical of Ruapehu (Fig. 4). Observations of persistent gas release confirmed the continued presence of magmatic degassing (Figs. S1, S2). However, given modelled heat flux increases of 62 MW per day (Fig. 4g; Behr et al. 2023), it is possible that magma ascent may have begun as early as March 10. The effect of stronger heat flux can be seen clearly in the temperature rise starting on 13 March. However, in the days prior to that, heat flux had probably already increased, but the effect on lake temperature was masked by an increase in cold water inflow. The initial heating of the lake may reflect the gas pushing up hot water in the hydrothermal system into the lake. The onset of tremor then occurred once a critical threshold for gas pressure was met, with a relatively dry pathway established for gas to pass through (Fig. 7a). Lags in lake temperature increases can also be explained by the latency of the lake system itself when undergoing convective heat transfer from vent sites to the outlet monitoring station. Alternatively, magma ascent did not occur during this preliminary stage, and heat flux increases may instead reflect the regulatory effect of liquid elemental sulphur, both in the cap and at the bottom of the crater lake, on heat input into the crater lake after exceeding its viscosity threshold.

#### Phase 1: 13–17 March

During Phase 1, we attribute heightened volcanic tremor to increased gas flow through the permeable cap due to ascending magma in the conduit degassing due to depressurisation, with gas accumulating beneath the cap (Fig. 7b). This influx of gas likely increased the pressure at the outflowing pathways, leading to increased gas flux and the observed tremor signals typical of this phase’s onset (Fig. 7b). Increased outgassing through the cap also resulted in convective heat transfer into the crater lake, causing an increase in lake temperatures (Fig. 4g). During this phase, we also observed the first drumbeats of the unrest episode. These drumbeats accelerated and decelerated (i.e., decreased and increased IETs) within a short ($\sim$3 h) window, suggesting they are unlikely to reflect transient changes in driving pressure and instead that the seal was not yet developed enough to be a stable fixture. Moreover, the coupled increase in IETs and amplitudes observed on March 16 indicate seal strengthening that resulted in longer loading cycles and increased energy release per failure (Fig. 4c, d).

**Fig. 8:**
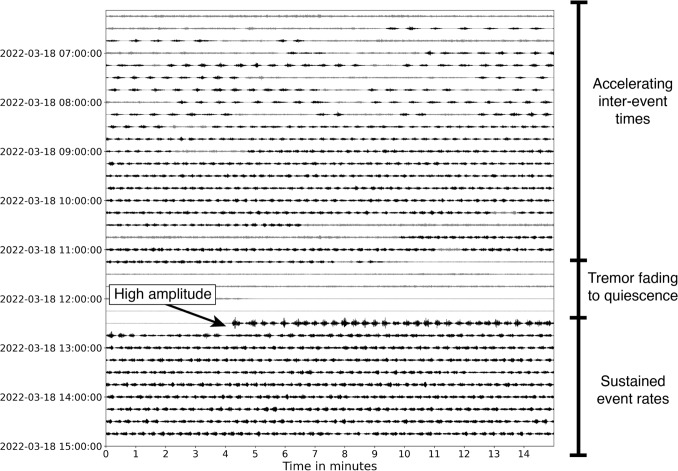
Waveform dayplot for March 18, 2022, on the vertical component of MAVZ, drumbeats are highlighted in black. Accelerating inter-event times (IETs) lead into a brief period of tremor that fades to quiet. Drumbeats then resume with high-amplitude events for the first 15 min before exhibiting sustained amplitudes and event rates. Data are bandpass filtered between 0.5 and 12 Hz

**Fig. 9:**
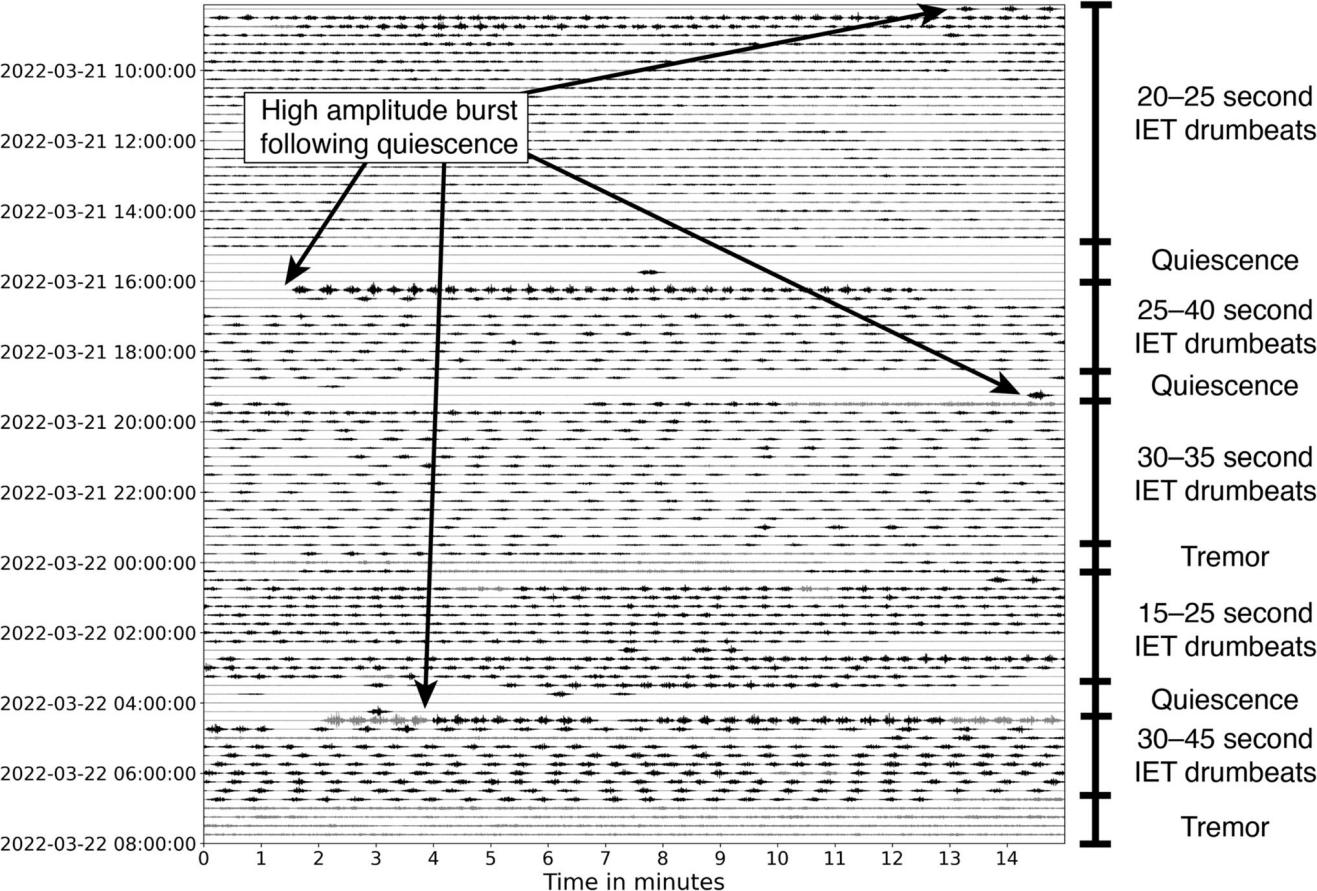
Waveform dayplot for Phase 2, March 21–22, 2022, on the vertical component of MAVZ, drumbeats are highlighted in black. Multiple drumbeat sets were observed during this period, each with their own IETs and separated by brief periods of quiescence or tremor. Sets that follow quiescence always initiate with higher-amplitude drumbeats. Data are bandpass filtered between 0.5 and 12 Hz

**Fig. 10:**
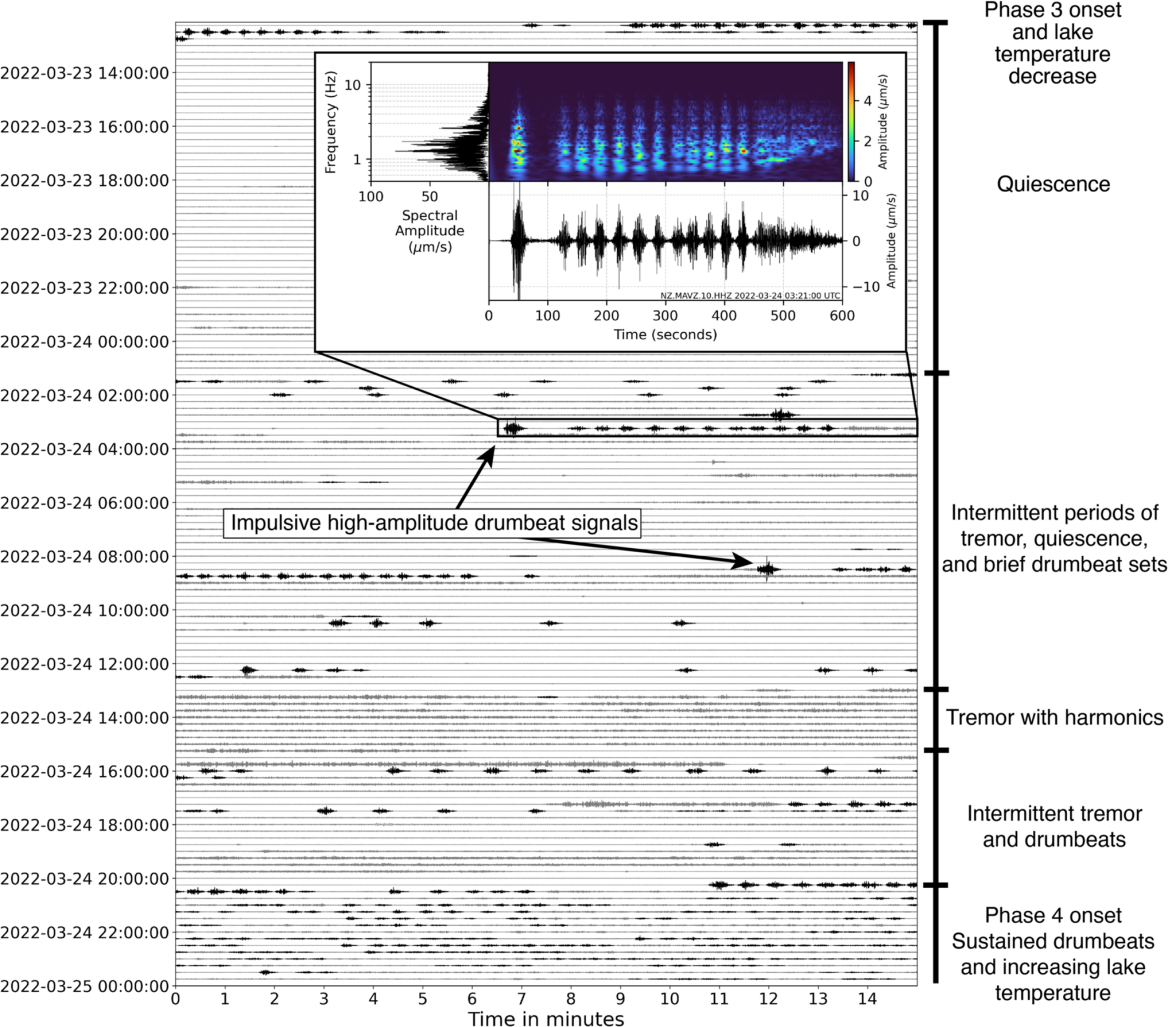
Waveform dayplot for March 23–24, 2022, on the vertical component of MAVZ, illustrating the entirety of Phase 3 and beginning of Phase 4, drumbeats are highlighted in black. Phase 3 commences with seismic quiescence for 12 h before sparse drumbeats and several high-amplitude, impulsive events occur. Tremor is present thereafter, followed by the reinitiation of sustained drumbeat generation in Phase 4. Highlighted section displays seismic power spectral density amplitude, frequency spectrogram, and seismogram of one of these high-amplitude, impulsive drumbeats and the events that followed. Dayplot data are bandpass filtered between 0.5 and 12 Hz, spectrogram between 0.5 and 20 Hz

**Fig. 11:**
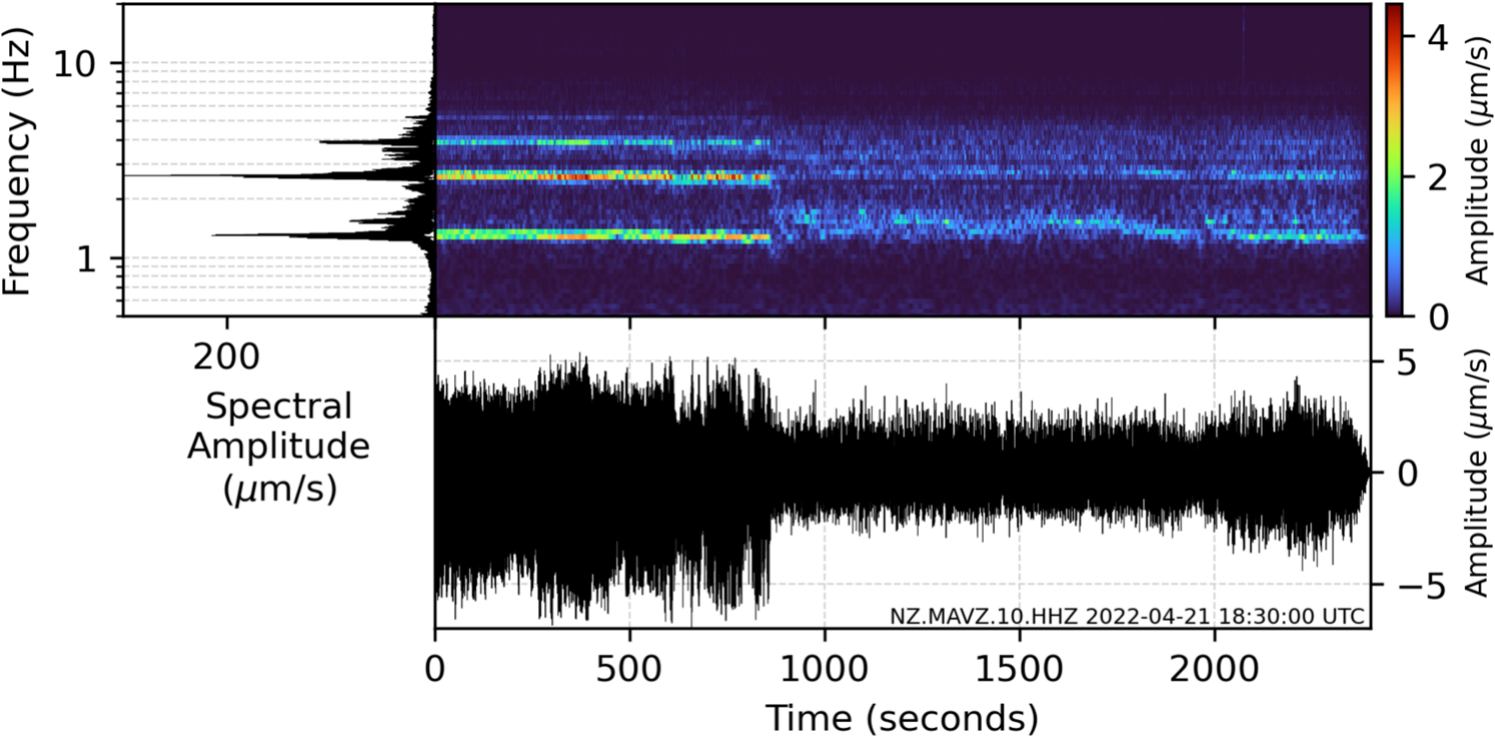
Power spectral density amplitudes, spectrograms, and seismograms for a section of volcanic tremor at 18:30:00 on April 21. An abrupt change from harmonic to broadband tremor is observed over a seconds-long timescale. Data are bandpass filtered between 0.5 and 20 Hz

#### Phase 2: 17–23 March

The onset of Phase 2 displayed complex source dynamics. The presence of consistent drumbeats indicates the establishment and regular failure of a permanent seal (Fig. 7c). Decreasing IETs alongside a sudden increase in event amplitudes in the first few hours of this phase suggest a coupled increase in both seal strength and loading rate over a short (minutes-scale) period (Fig. 8).

Phase 2 also possessed multiple 1–2-h bursts of high-amplitude drumbeats following short periods of quiescence (Fig. 8). We suggest they represent forceful seal failure events as a result of excess pressure accumulation from a temporary increase in seal strength. This explains periods of quiescence, caused by the seal heavily restricting outgassing, and the higher (up to 10 times) amplitude signals that followed (Fig. 7c). During Phase 2’s final 2 days of activity, multiple drumbeat sets were observed with brief (1–2 h) interspersed periods of quiescence and tremor. This behaviour was unique for the 2022 unrest in that each set displayed its own range of IETs (Figs. 4c, 9), and many often displayed higher amplitudes when following quiescence. They also displayed distinct patterns of either short-IET/high-amplitude signals or long-IET/high-amplitude signals (Figs. 4c, d, 9). We explain this highly dynamic behaviour through temporary changes in seal strength and the rate of magmatic degassing.

#### Phase 3: 23–24 March

The onset of Phase 3 was the most seismically quiescent period of the entire unrest. This period was interspersed with occasional low-amplitude tremor signals and sparse drumbeats followed by several much larger amplitude, impulsive signals (Fig. 10). We suggest that the initial 12 h of Phase 3 represent significant seal strengthening and little to no gas outflow was occurring (Fig. 7d). This is supported by the sudden decrease in lake temperature and heat flow (Behr et al. 2023) with the onset of Phase 3 (Fig. 4g). Moreover, the highly variable behaviour observed in the latter stages of Phase 2, as well as the observed quiescence at the onset of Phase 3, suggests significant seal strengthening at the Phase 2/Phase 3 boundary that largely reduced gas and heat transfer to the crater lake. This seal then forcefully failed and released pressure at several points in the following hours, as shown by the occurrence of high-amplitude, impulsive signals (Fig. 7d). These seal failures would have allowed intermittent gas and heat release, while also decreasing gas driving pressures going into Phase 4.

In hindsight and after the detailed study presented here, the seismic and heat flux signals suggest the 2–3 days surrounding the Phase 2/Phase 3 boundary was when the likelihood of eruption was highest. In the final stages of Phase 2, multiple instances of strong sealing and intermittent periods of drumbeat sets were observed; indicating that outgassing was primarily obstructed and magmatic degassing was increasing. That seal strength appeared to be increasing throughout this period while degassing and gas cavity driving pressures were also increasing indicates conditions were mostly or even entirely preventing outgassing. A nearly complete blockage occurred at the onset of Phase 3, when no seismic signals were observed and heat flux to the surface rapidly declined (Figs. 4, 10). It is during this 12-h period in Phase 3 that we suggest an eruption was most likely to occur if pathway blockage had continued. However, high-amplitude impulsive events suggest that the seal failed and outgassing resumed, precluding an eruption.

#### Phase 4: 24 March–5 April

Phase 4 is one of the most stable phases of the 2022 unrest. A cycling of high-amplitude drumbeat sets and quiescence occurs initially, suggesting that the seal, while still relatively strong in relation to driving pressure, experienced gradual weakening during the first $\sim$18 h of this phase. Following this, the acceleration of drumbeats to sustained IETs and amplitudes indicates that either seal strength decreased to the point of equilibrium with the system, or degassing and pressure increased to maintain regular loading/failure cycles. Lake temperatures and heat flux both increase in accordance with increased heat and gas transfer to the surface (Fig. 7e). Phase 4 also featured significant increases in drumbeat amplitude without any accompanying change in IET, particularly during the final 8 days of Phase 4 (Fig. 4). We propose that these changes were caused by a shallowing of the drumbeat source and thus a decrease in source-receiver distance (Fig. 7e).

#### Phase 5: 5–9 April

Discrete drumbeat signals are observed to linearly accelerate and to form continuous tremor at the onset of Phase 5, a behaviour that we ascribe to coupled changes in both seal mechanics and the degassing regime (Fig. 7f). Notably, during this phase, we also observe a plateauing of lake temperatures and significant decrease in heat input which, given the presence of tremor, we suggest represents decreased degassing and heat release into the lake (Fig. 7f). However, a decrease in the seal’s strength must also have occurred in order for the ascent of gas to continue through the pathway unimpeded. We suggest this coupled change occurs due to the decreased supply of sealant material from depth given decreased gas flux. Tremor frequencies also varied significantly during this period. Central frequencies experienced a sudden increase of 0.5 Hz at the onset of this phase, and harmonics appeared in the spectra that changed dominant frequencies until April 7 before returning to near-broadband signals. We attribute these changes to a shallowing of the oscillation location and/or changes in the oscillation geometry subsequent to the degradation of the seal (Fig. 7f). This explains not only the increase of central frequencies due to the decreased effect of attenuation, but also the change in source location allowing harmonic resonance where previously none was present.

#### Phase 6: 9–12 April

Phase 6 marked the re-establishment of sealing, albeit in a different manner to previous episodes of sustained drumbeats (Fig. 7g). Initially, drumbeats were interposed with low-amplitude tremor and overlapping drumbeat signals that were faintly harmonic in similar spectral bands as those in Phase 5. Signals then gradually transitioned into drumbeats only (Fig. 4). We suggest this behaviour resulted from a seal being gradually re-established until continuous outgassing was stalled and loading/failure cycles resumed. This may have been the result of increased degassing and sealant material transfer and/or the gradual build up of sealant material. A brief period of continuous gas flow, as indicated by tremor signals, was then followed by a sudden increase in both IET and amplitude (Fig. 4). A similar pattern was observed in Phase 1, and indicates strengthening of the seal resulting in increased energy release after longer loading cycles. Over the course of April 11 IETs and amplitudes both decreased, suggesting a gradual weakening of the seal (Fig. 7g).

#### Phase 7: 12 April–8 May

Following the cessation of drumbeats at the end of Phase 6, several hours of quiet ensued before a high-amplitude event occurred similar to those observed in Phase 3 (Fig. 3). This event may also represent forceful seal failure and rapid depressurisation that altered source geometries enough to maintain a clear pathway for the remainder of the unrest (Fig. 7h). The lack of drumbeats throughout Phases 7 and 8 may be explained by a decrease in sealing material being transported through the pathway or, more likely, increased outgassing being facilitated by altered pathway geometries. New geometries were varied at first, as evidenced by the several hours of banded and spasmodic tremor (e.g., Konstantinou and Schlindwein 2003; Lesage et al. 2006; Arámbula-Mendoza et al. 2016) following this event, however these ultimately settled and allowed continuous tremor and gas flux through the system.

Phase 7 featured sustained heat flux, increasing lake temperatures, and two measurements of elevated gas flux (Fig. S2), further suggesting continuous elevated gas and heat transfer (Fig. 3g). Harmonic tremor with similar spectral peaks to that observed in Phase 5 was maintained throughout much of Phase 7 (Fig. 11), indicating a similar source location. This harmonic tremor has similarities to observations of ‘chugging’ from gases flowing through partially constricted channels following explosions at Karymsky volcano (Lees et al. 2004); however, we did not observe any explosive or acoustic activity at Ruapehu. Slight fluctuations in tremor amplitude and accompanying spectral gliding were also observed throughout. In almost all cases, spectral gliding occurred as an increase in frequency with a corresponding decrease in tremor amplitude, and a decrease in frequency when tremor amplitude increased. Spectral gliding has often been observed during volcanic activity around the world, most commonly in relation to eruptive activity (e.g., Neuberg 2000; Jousset et al. 2003; De Angelis and McNutt 2007; Lees et al. 2008; Maryanto et al. 2008; Hotovec et al. 2013; Unglert and Jellinek 2015). Gliding has been explained by varying rates of overlapping repeating earthquakes (Neuberg 2000; Hotovec et al. 2013), changes in resonator dimensions (e.g., gas pocket above a magma column; De Angelis and McNutt 2007; Maryanto et al. 2008), or variations in gas flow (Lees et al. 2008; Unglert and Jellinek 2015). However, modelling of harmonic tremor finds that decreases in seismic amplitudes with simultaneous frequency gliding may be indicative of sealing (Girona et al. 2019). Therefore, we suggest that the source of gliding tremor was slight constrictions in the fluid pathway due to sulphur/mineral precipitation (Fig. 7h). During this period, a 1.5-km tall steam plume was observed above the summit region (GeoNet 2022d); however, the lack of accompanying seismic signals indicates that this is not tied to any strong activity and is instead likely the result of increased heat flow and cold atmospheric conditions.

#### Phase 8: 8 May–19 June

The final phase of the 2022 unrest episode, Phase 8, marks the gradual decrease of degassing as magma cools and/or receives no new injection into shallow storage reservoirs. This caused gas cavity size and, consequently, the rate of outgassing through the pathway to decrease (Fig. 7h). The gradual decrease in both tremor presence and frequencies to background microseism levels supports this, as well as the decline in both lake temperature measurements and calculated heat flux over the 42 day period, albeit with a short-lived increase in heat flux at the end of this period (Fig. 3g).

### Implications for future monitoring

This research has the potential to contribute to future volcanic monitoring practices in several ways. This is the first detailed documentation of drumbeat earthquakes and tremor during an unrest event at Ruapehu. As well as the improved understanding of the 2022 unrest event, it is highly likely that similar behaviour will be observed during future unrest events. This gives important context for interpretation and eruption forecasting in the future. We also hope that by making the manually created catalogue of drumbeat earthquakes and tremor publically available, this can serve as a resource for the development of more automated analysis approaches, such as template-matching (e.g., Wimez and Frank 2022; Tan et al. 2023).

Much could be added to Ruapehu’s current monitoring network that would benefit future analysis and hazard assessment should similar signals to those observed in 2022 be seen again. Several observations of sediment upwelling in the crater lake from both the northern and central vents were published during the 2022 unrest (Fig. S2; GeoNet 2022c, d, e). Some changes in upwelling potentially coincided with changes in the seismic regime, though interpretations from this are limited given the low time resolution of observations. While real-time camera monitoring of the crater lake and any potential precursory phenomena would benefit multidisciplinary analyses and hazard assessment, we recognise the importance of Ruapehu and Te Wai ā-moe to Ngāti Rangi (Gabrielsen et al. 2018; Pardo et al. 2015). Te Wai ā-moe is a place of immense inter-generational cultural importance to Ngāti Rangi, and they are its kaitiaki (guardians). Therefore, any monitoring of Te Wai ā-moe must continue to be done alongside them.

One further limitation to this work was the low temporal resolution of gas composition and flux monitoring data (monthly) compared with seismic monitoring data. Phases 1, 2, 3, and 5 contain no gas measurements, while Phases 4 and 6 contain one for each primary gas type, ineffective for comparative analysis with other monitored signal types (Figs. S1, S2, GNS Science 1954). Continuous SO$_2$ flux measurements using ScanDOAS was only implemented towards the end of Phase 7 in early May, 2022 (GNS Science 2022). During future unrest at Ruapehu, ScanDOAS sensors will no doubt assist data interpretation by measuring the short-term variability of gas flux at Ruapehu. However, the effect of sulphur scrubbing by the crater lake and the effect it has on SO$_2$ flux should be considered carefully in future analyses (Symonds et al. 2001; Hughes et al. 2024; Christenson 2000; Christenson et al. 2010; Werner et al. 2006; Christenson 1994). Conversely, CO$_2$ is largely unaffected by lake scrubbing processes (Harris and Rose 1996; Symonds et al. 2001), and its use would be amplified by its own form of continuous, high-temporal resolution monitoring (e.g., Multi-GAS; Aiuppa et al. 2005; Shinohara 2005). This would also enable measurements of the ratios of CO$_2$, SO$_2$, and H$_2$S, and could potentially be used to compliment seismic and lake temperature measurements at Ruapehu (de Moor et al. 2016; Roberts et al. 2017; Moune et al. 2022; Taylor-Offord et al. 2023).

## Conclusions

The 2022 unrest episode at Ruapehu provides new insights into the state of Ruapehu’s magmatic-hydrothermal system and offers new information regarding drumbeat earthquake processes and shallow conduit dynamics. We present a seismic catalogue with associated analyses for the 2022 unrest period. We manually picked 43,000 discrete drumbeat events, 6 days of overlapping drumbeats, and 89 days of volcanic tremor and analysed temporal distributions and signal characteristics. We find that the majority of events displayed inter-event times of 20–25 s and central frequencies of 1.8–2.3 Hz. A timeline of the 2022 unrest period’s evolution outlines eight phases of activity where signal characteristics and source processes changed significantly. Our study allows us to propose the following findings and interpretations of seismic, lake temperature, and other signals observed during Ruapehu’s 2022 unrest episode.

- The system governing this unrest period consists of shallow magma storage and ascent within the conduit, a cavity of volcanic gas overlying shallow magma, a permeable cap with fracture pathways, and the overlying crater lake.
- Drumbeat and volcanic tremor signals arose from the intermittent emplacement of a seal within outgassing pathways. Drumbeats were the result of periodic seal loading/failure cycles driven by gas cavity pressures, while unimpeded pathways and gas flow oscillation resulted in tremor signals.
- The seal composition likely consists of an assemblage of sulphur and sulphate minerals.
- The presence of all seismic signals correlates temporally with changes in crater lake temperatures. Where inferred outgassing is occurring through the permeable pathway (i.e., drumbeats and tremor are being produced) lake temperatures are also observed to increase, suggesting gas and heat were transferred to the surface.
- An eruption during the 2022 unrest period likely did not occur due to pathway geometries being able to facilitate large enough volumes of gas flow and/or the seal being too newly developed to maintain material strength and create sustained blockages of outgassing pathways.
- Future monitoring at Ruapehu could be improved by continuous visual monitoring of the crater lake Te Wai ā-moe and the installation of additional permanent gas monitoring stations.

In the case of future seismic unrest, these observations will help to rapidly assess the significance of the seismicity being recorded. During periods of volcanic unrest, understanding of the internal source processes present during previous unrest episodes can help to grant more informed judgements of ongoing source processes. From this, hazard and risk assessments are better informed and can ensure that local communities, visitors, and governments are advised of potential dangers, safeguarding livelihoods and mitigating future harm.

## Supplementary Information

Below is the link to the electronic supplementary material.

Supplementary file 1 (pdf 294 KB)

## Data Availability

The full catalogue of different seismic events described in this study is available at https://doi.org/10.5281/zenodo.13910455. The GeoNet seismic data is freely available through GeoNet (GNS Science 2021), as is the crater lake temperature (GNS Science 2018). Figures were made with GMT6 (Wessel et al. 2019), Matplotlib (Hunter 2007), and PyGMT (Tian et al. 2023).
