# Supplementary material for: Source dynamics of Ruapehu’s 2022 volcanic unrest: insights from drumbeat seismicity, tremor, and crater lake signals
Source: Bull Volcanol. 2025 May 19;87(6):44. doi: 10.1007/s00445-025-01823-2 (PMC12089182; doi:10.1007/s00445-025-01823-2)
Supplement: Supplementary file 1 — (pdf 294 KB) [file 445_2025_1823_MOESM1_ESM.pdf]

Supporting Information for "Source dynamics of Ruapehu's 2022 volcanic unrest: Insights from drumbeat seismicity, tremor, and crater lake signals"

L. Bramwell[1], F. Illsley-Kemp[1], E. Hughes[2], S. Butcher[3], O.D. Lamb[4], Y. Behr[4]

[1]School of Geography, Environment and Earth Sciences, Victoria University of Wellington, Wellington, New Zealand

[2]Te Pū Ao | GNS Science, Avalon Campus, Lower Hutt, New Zealand

[3]British Geological Survey, Edinburgh, United Kingdom

[4]Te Pū Ao | GNS Science, Wairakei Research Centre, Taupō, New Zealand

Contents of this file

1. Figure S1
2. Figure S2
3. Table S1
4. Table S2

Introduction

This supplementary information contains two figures, showing gas flux variations and observations at Ruapehu, and two tables, summarising the observations presented in this paper and station characteristics.

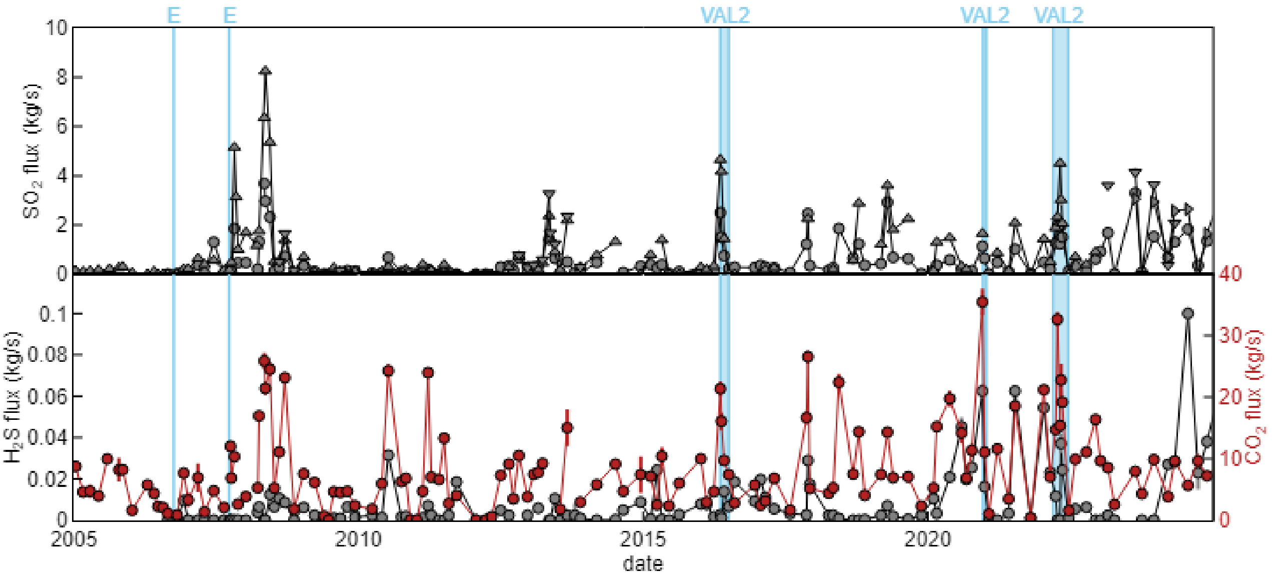

Figure S1. Long-term gas flux measurements using an airborne platform at Ruapehu by GeoNet since 2005 (GNS Science, 1954). The top panel shows SO_2 flux measured using different methods: contouring (circle), cospec (up triangle), flyspec (down triangle), and mobile-doas (right triangle). The bottom panel shows CO_2 (red, right-hand axis) and H_2S (grey, left-hand axis) flux measured using contouring. The blue vertical lines indicate eruptions (E) and periods of moderate-to-heightened unrest (VAL2) (GNS Science, 1994).

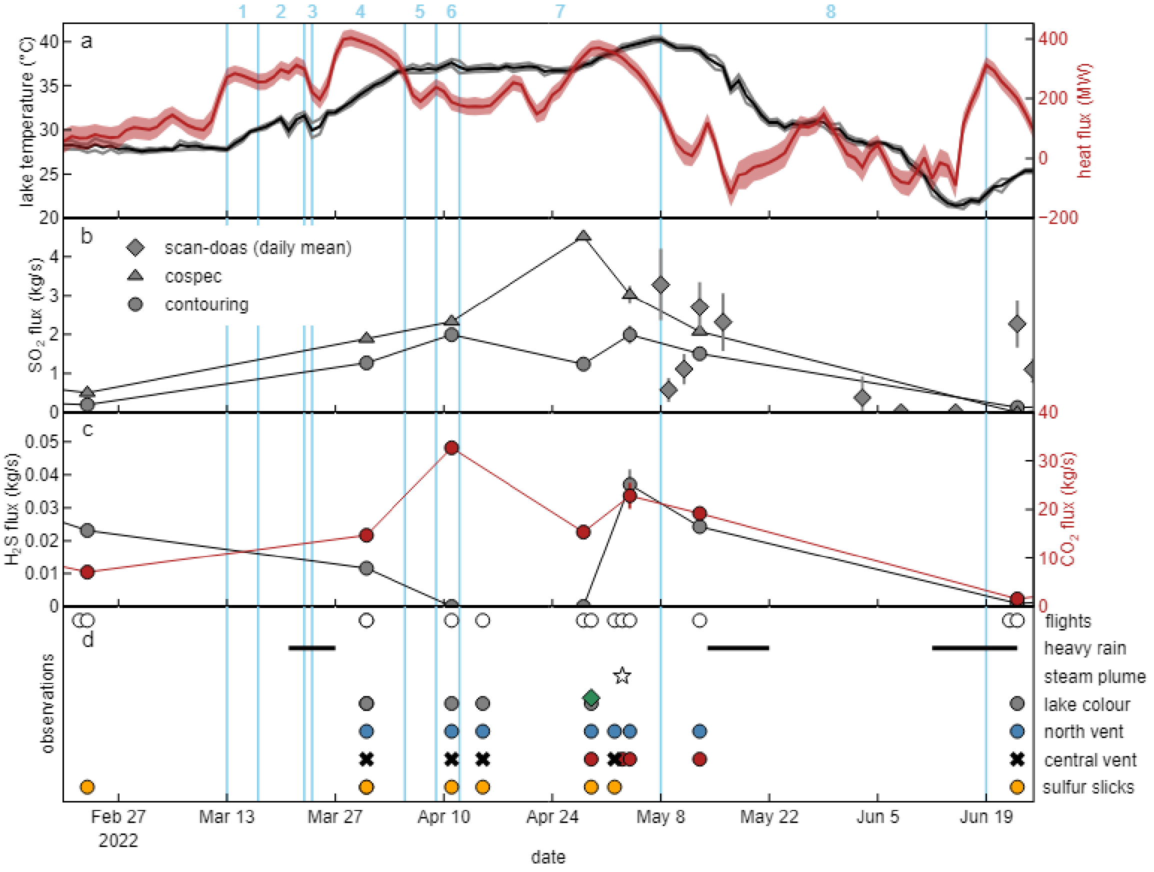

Figure S2. Gas flux and observations of Ruapehu during the 2022 unrest. (a) Daily average lake temperature (black, left-hand axis; GNS Science, 2018) and heat flux (red, left-hand axis; Behr et al., 2023), with associated uncertainty. (b) SO_2 flux measured using contouring (circle), cospec (up triangle), and scan-doas (daily average, diamond) (GNS Science, 1954, 2022). (c) CO_2 (red, right-hand axis) and H_2S (grey, left-hand axis) flux (GNS Science, 1954). (d) Observations of Te wai ā-moe from VABs (GeoNet, 2022b, 2022e, 2022f, 2022g, 2022h, 2022n, 2022a, 2022c, 2022d, 2022i, 2022j, 2022k, 2022l, 2022m): white circles show when flights were conducted over the lake; black horizontal lines when heavy rain was noted; white star indicates a steam plume; lake colour is indicated by a grey circle for 'battleship grey' or green diamond for blue/green areas; a coloured circle indicates the presence of north vent (blue), central vent (red), or sulfur slicks (yellow), a cross means the absences of the feature was noted, and no symbol means that feature was not mentioned (but does not necessarily mean the feature was not present).

Table S1: Timeline summary of Ruapehu's 2022 unrest period. Figures and sources in text.

Date	Description
Pre-Phase 1 (up to March 13, 2022)	
To March 13	Background levels of seismicity, stable crater lake temperatures, and increase in heat flux from March 10.
Phase 1 (March 13–17, 2022)	
March 13	Gradual increase in volcanic tremor intensity, crater lake temperatures begin increasing.
March 14	First set of drumbeats from multiple families.
March 15	Very low amplitude and frequency drumbeats.
March 16	Drumbeats with coupled increase in IETs and amplitudes. Variable discretisation into overlapping drumbeat/tremor signals.
Phase 2 (March 17–23, 2022)	
March 17	Sustained drumbeat generation (Group B families most common), crater lake temperatures continue to increase.
March 18-23	Multiple bursts of high amplitude drumbeats following brief periods of quiescence.
March 21	Decrease in lake temperature during heavy rainfall event, heat flux increases. Distinct sets of drumbeats with differing IETs and dominant families interposed with tremor. VAL raised to Level 2.
March 22	Increasing lake temperature and sustained heat flux.
Phase 3 (March 23–24, 2022)	
March 23	Seismic quiescence, decreasing lake temperatures and heat flux, moderate rainfall event.
March 24	Sporadic drumbeat sets with multiple VT-like high amplitude signals, tremor more prominent.
Phase 4 (March 24–April 5, 2022)	
March 24–25	Drumbeats resume in sets interposed with periods of quiescence or low amplitude tremor, crater lake temperatures and heat flux begin increasing.
March 25–28	Sustained drumbeat generation and event rates.
March 28	Step-wise increase in drumbeat amplitude and frequency follow brief tremor episode.
March 29–31	Several short bursts of high amplitude drumbeats, heat flux begins to decrease while lake temperatures continue increasing.
March 31	Multiple sulphur slicks observed above the northern vent region of the lake.
April 2	Second step-wise increase in drumbeat amplitude occurs in two parts. Group C drumbeats most common.

April 3–5	Drumbeat amplitudes decrease with slight fluctuations, heat flux begins declining rapidly.
Phase 5 (April 5–9, 2022)	
April 5	Linear acceleration of discrete drumbeats to overlapping drumbeat then continuous harmonic tremor signals with higher central frequencies than previously observed. Shift in peak spectra. Lake temperatures and heat flux plateau.
April 6	Change in peak frequencies. Irregular spectral gliding.
April 6–7	Sparse drumbeat and overlapping drumbeat signals.
April 7	Mostly broadband tremor spectra with faint harmonics.
April 7–9	Minor spectral gliding during tremor and drumbeat signals.
Phase 6 (April 9–12, 2022)	
April 9	Reinitiation of discrete and overlapping drumbeat signals, though tremor largely dominates. Bursts of high amplitude tremor present. Crater lake temperatures begin increasing slowly.
April 10	Exclusively drumbeat signals with coupled increase in IET and amplitude. Occasional harmonic tremor episodes.
April 11	Decrease in drumbeat IET, amplitude, and frequency.
April 12	Significant increase in drumbeat IET with no change in other metrics.
Phase 7 (April 12–May 8, 2022)	
April 12	Initially quiescent. Single, impulsive high amplitude event followed by broadband banded and spasmodic tremor that developed harmonics through time.
April 13–21	Moderate to strong harmonic tremor. Multiple upwellings and sulphur slicks observed from northern vent region.
April 21	Abrupt change from strong harmonics to broadband tremor signals with faint harmonics.
April 22	Heat flux begins increasing.
April 23	Tremor decelerates to form discrete drumbeat set, with variable acceleration/deceleration into and out of tremor for the set's duration. Broadband tremor continues thereafter with occasional harmonics.
April 28	Lake temperatures begin increasing.
April 29–May 2	Several periods of tremor transitioning to multiple-hour-long quiescence. Spectral gliding entering and leaving these periods.
May 2	Heat flux begins declining. Upwelling observed from the central vent for the first time, decreased upwelling from the northern vent.
May 3	Steam plume observed.
Phase 8 (May 8–June 19, 2022)	
May 8	Brief period of quiescence transitioning to small drumbeat sequence that accelerates into harmonic tremor. Lake temperatures begin declining.

May 8–22	Broadly decreasing tremor amplitudes and frequencies. Upwelling of the central and northern vents observed on two occasions.
May 22	Brief period of quiescence and increasing tremor amplitudes.
May 23–June 3	Broadly decreasing tremor amplitudes and frequencies.
June 3	Brief drumbeat set with interposed periods of overlapping drumbeats.
June 3–17	Tremor with amplitudes slightly above background noise.
June 17	Variable banded, harmonic, and broadband tremor signals with sparse drumbeat events.
June 18	Drumbeats accelerate into continuous harmonic tremor, which is soon followed by periods of banded, harmonic, and spasmodic tremor signals before quiescence ensues.
Post Phase 8 (June 19–July 4, 2022)	
June 19–July 4	Very low amplitude and frequency tremor at near-background levels. No observed drumbeats. Plateaued lake temperatures and slightly fluctuating heat flux.
July 4	VAL lowered to Level 1.

Table S2. Details on GeoNet stations used for this study. All sites recorded data at 100 samples per second using a Kinemetrics Quanterra Q330S+ digitiser, except for WHVZ which used a Nanometrics Centaur CTR4-6S. Details are accurate for April 2022 (GNS Science, 2019).

Name	Longitude	Latitude	Altitude (m a.s.l)	Type	Seismic Sensor
COVZ	175.542	-39.200	1133	BB-BH	Nanometrics Trillium Compact 120 PH-2
ETVZ	175.711	-39.136	1236	BB	Guralp CMG-3ESPC
FWVZ	175.553	-39.255	2043	BB	Guralp CMG-3ESPC
KRVZ	175.641	-39.094	1207	SP	Sercel L4C-3D
MAVZ	175.562	-39.268	2624	BB-BH	Guralp CMG-3ESPC
MOVZ	175.753	-39.407	874	SP	Sercel L4C-3D
MTVZ	175.470	-39.385	840	SP	Sercel L4C-3D
NGZ	175.601	-39.176	1452	SP	Sercel L4C-3D
NOVZ	175.611	-39.126	1476	SP	Lennartz Electronic LE-3DliteMkII
NTVZ	175.676	-39.098	1260	BB	Guralp CMG-3ESPC
OTVZ	175.665	-39.163	1506	BB	Guralp CMG-3ESPC
SNVZ	175.640	-39.186	1604	SP	Lennartz Electronic LE-3DliteMkII
TMVZ	175.704	-39.116	1200	BB	Nanometrics Trillium 120QA
TRVZ	175.548	-39.299	2062	BB	Guralp CMG-3ESPC
TUVZ	175.654	-39.268	1446	SP	Sercel L4C-3D
TWVZ	175.438	-39.071	1084	SP	Sercel L4C-3D
WHVZ	175.589	-39.282	2105	BB	Guralp CMG-3ESPC
WNVZ	175.598	-39.327	1566	SP-BH	2Hz Duke Malin
WTVZ	175.590	-39.115	1186	SP	Sercel L4C-3D

BB: Broadband, SP: Short-period, BH: Borehole installation

References

Behr, Y., Sherburn, S., & Hurst, T. (2023). Continuous estimates of heat emission at Mt. Ruapehu using the Unscented Kalman Smoother. *Journal of Applied Volcanology*, *12*(1), 1–10. doi: 10.1186/s13617-022-00125-y

GeoNet. (2022a). Volcanic Activity Bulletin RUA - 2022/03: Strong tremor and Crater Lake heating continues at Mt Ruapehu. Volcanic Alert Level remains at Level 2. Retrieved from https://www.geonet.org.nz/vabs/7dyfMjTIP8ITRFrv1kmlL0

GeoNet. (2022b). Volcanic Activity Bulletin RUA - 2022/04: Mt Ruapehu volcanic tremor and Crater Lake heating continues. Volcanic Alert Level remains at Level 2. Retrieved from https://www.geonet.org.nz/vabs/35MAcvBHcMYJn9q81yrRJw

GeoNet. (2022c). Volcanic Activity Bulletin RUA - 2022/05: Mt Ruapehu elevated volcanic unrest continues. Volcanic Alert Level remains at Level 2. Retrieved from https://www.geonet.org.nz/vabs/78BGTZnjgWVgRIWY20Jfo1

GeoNet. (2022d). Volcanic Activity Bulletin RUA - 2022/07: Mt Ruapehu strong volcanic tremor continues. Volcanic Alert Level remains at Level 2. Retrieved from https://www.geonet.org.nz/vabs/5bBmZ1XGPcV1oNfatla5bk

GeoNet. (2022e). Volcanic Activity Bulletin RUA - 2022/08: Volcanic unrest continues at Mt Ruapehu marked by increased volcanic gas output and strong volcanic tremor: Volcanic Alert Level remains at Level 2. . Retrieved from https://www.geonet.org.nz/vabs/7DdT7UXjoGgxKswkoo2cte

GeoNet. (2022f). Volcanic Activity Bulletin RUA - 2022/09: Steam plume visible at Mt Ruapehu, volcanic unrest continues: Volcanic Alert Level remains at Level 2. Retrieved from https://www.geonet.org.nz/vabs/3BdNUMuBwVuzqejjXPbQd4

GeoNet. (2022g). Volcanic Activity Bulletin RUA - 2022/10: Heightened volcanic unrest continues at Mt Ruapehu with volcanic gas output, strong tremor and steam plumes: Vol-

canic Alert Level remains at Level 2. Retrieved from https://www.geonet.org.nz/vabs/1q6vYluM1u0yiWyVXsA2NR

GeoNet. (2022h). Volcanic Activity Bulletin RUA - 2022/11: Volcanic tremor at Mt Ruapehu lowers from strong to moderate: Volcanic Alert Level remains at Level 2. Retrieved from https://www.geonet.org.nz/vabs/36DmsaLzVWczmz5uyAs71p

GeoNet. (2022i). Volcanic Activity Bulletin RUA - 2022/12: Volcanic unrest continues at Mt Ruapehu as gas emissions remain elevated. The Volcanic Alert Level remains at Level 2. Retrieved from https://www.geonet.org.nz/vabs/5IpA3XJE5VXLlXHXqeFlc

GeoNet. (2022j). Volcanic Activity Bulletin RUA - 2022/13: Mt Ruapehu volcanic unrest continues with moderate tremor and a cooler Crater Lake. The Volcanic Alert Level remains at Level 2. Retrieved from https://www.geonet.org.nz/vabs/4s14hjflDtLovxBQAFL9ks

GeoNet. (2022k). Volcanic Activity Bulletin RUA - 2022/14: Lower level of activity at Mt Ruapehu. The Volcanic Alert Level remains at Level 2. Retrieved from https://www.geonet.org.nz/vabs/VC7MzZKnEyWtYemJSrFvz

GeoNet. (2022l). Volcanic Activity Bulletin RUA - 2022/15: Moderate volcanic unrest continues at Mt Ruapehu. The Volcanic Alert Level remains at Level 2. Retrieved from https://www.geonet.org.nz/vabs/7zjgIz7w7KOzC7RPwYb3J5

GeoNet. (2022m). Volcanic Activity Bulletin RUA - 2022/17: Ruapehu Crater Lake sampled, variable gas emissions and low volcanic tremor levels continue. The Volcanic Alert Level remains at Level 2. Retrieved from https://www.geonet.org.nz/vabs/55YI3w8BfAESEqtjmUVa7R

GeoNet. (2022n). Volcanic Activity Bulletin RUA - 2022/18: Level of unrest continues to decrease at Mt Ruapehu. The Volcanic Alert Level is lowered to 1. Retrieved from https://www.geonet.org.nz/vabs/6ek5OkTf13CM9BqY538frb

GNS Science. (1954). *GeoNet Aotearoa New Zealand Manually Collected Volcano Data [Data*

set]. doi: 10.21420/PSP7-KW60

GNS Science. (1994). *GNS Science Aotearoa New Zealand Volcanic Alert Level Datasets [Data set].* doi: 10.21420/we5s-1n52?x=y

GNS Science. (2018). *GeoNet Aotearoa New Zealand Automatically Collected Volcano Data [Data set].* doi: 10.21420/EN0F-XY29

GNS Science. (2022). *GeoNet Aotearoa New Zealand ScanDOAS Data [Data set].* doi: 10.21420/HC1T-QJ63
